# Development and preliminary validation of an AI acceptance scale for intercultural teaching and its cross-sectional associations with university teachers’ occupational wellbeing

**DOI:** 10.3389/fpubh.2026.1986187

**Published:** 2026-10-28

**Authors:** Qi Zhang, Ramzy Mohammad Ismath, Abd Razak Bin Zakaria

**Affiliations:** Department of Educational Foundations and Humanities, Faculty of Education, Universiti Malaya, Kuala Lumpur, Malaysia

**Keywords:** AI acceptance, emotional exhaustion, intercultural teaching, job demands-resources model, occupational wellbeing, scale development, teacher self-efficacy

## Abstract

**Background:**

University teachers’ occupational wellbeing and emotional exhaustion are relevant occupational-health concerns in higher education. As generative artificial intelligence becomes embedded in teaching work, AI acceptance may represent a perceived, context-specific appraisal of whether AI is usable for intercultural teaching tasks. This study developed a preliminary measure of AI acceptance for intercultural teaching and examined its cross-sectional associations with teacher resources and occupational-health indicators.

**Methods:**

This two-sample cross-sectional study used Sample A to examine the empirical behavior of the AI Acceptance for Intercultural Teaching Scale and Sample B to test measurement validity and occupational health associations. IBM SPSS Statistics 27.0.1 was used for descriptive statistics, EFA, Cronbach’s alpha, correlations, and regression analyses. CFA and SEM were conducted in R 4.6.1 (lavaan 0.6.21, lavaan.mi 0.1.0, and mice 3.19.0) using robust maximum likelihood (MLR) estimation; CR and AVE were calculated from standardized CFA loadings, and HTMT from item correlations. Hypothesis tests used non-imputed scale-score regressions; multiple imputation (mice, *m* = 5) with D2-pooled CFA/SEM model tests served as sensitivity analysis, and indirect-association bootstrap intervals were sensitivity summaries rather than formal D2-pooled tests.

**Results:**

The AITA items were suitable for factor analysis in Sample A (KMO = 0.940), but the exploratory loading pattern did not cleanly reproduce the theorized four TAM-based dimensions. In Sample B, the overall AITA score showed good internal consistency (alpha = 0.858), whereas the four subdimensions showed substantial overlap and weaker reliability and convergent-validity evidence. AI acceptance was positively associated with intercultural teaching self-efficacy and autonomous teaching motivation. Self-efficacy was associated with both occupational wellbeing and lower emotional exhaustion, whereas autonomous motivation was associated primarily with occupational wellbeing. Direct associations between AI acceptance and the occupational-health outcomes were not significant.

**Conclusion:**

The present findings provide preliminary evidence for using the overall AITA score as a context-specific measure of AI acceptance in intercultural teaching. The results also show cross-sectional associations between AI acceptance and teacher-resource variables, but they do not establish causal effects, mediation, or intervention effectiveness. The four TAM-based dimensions should be treated as provisional content facets requiring further validation.

## 1 Introduction

University teachers’ occupational wellbeing and emotional exhaustion should be understood not only as individual work experiences, but also as public and occupational mental health concerns within higher education. Persistent emotional exhaustion can weaken teachers’ capacity to sustain teaching quality, contribute to intentions to leave the profession, and reduce the resilience of higher education systems. From this perspective, identifying modifiable resources that may help teachers adapt to changing teaching conditions is a public health-relevant task, especially when technological change is rapidly reshaping the organization of academic work.

Generative artificial intelligence (AI) is one such changing work condition. AI tools are rapidly entering higher education teaching and are increasingly involved in course design, teaching-resource generation, language support, learning feedback, and classroom interaction. In intercultural or internationalized teaching, these tools may support multilingual communication, intercultural case generation, international student support, preparation of culturally comparative materials, and online international collaboration. At the same time, AI may introduce uncertainty, ethical concerns, additional skill demands, and pressure to redesign teaching practices. AI acceptance is therefore not only a technology-adoption construct; it may also indicate whether teachers perceive AI as manageable and useful in their work.

Public health and occupational health literature increasingly recognizes that teacher wellbeing, burnout, and work resources are connected to the functioning and sustainability of education systems (1, 2). The Job Demands-Resources (JD-R) model provides a useful occupational health framework because it links job demands and resources with wellbeing, engagement, and burnout-related outcomes (3, 4). The Technology Acceptance Model (TAM) remains useful for measuring teachers’ AI acceptance through perceived usefulness, perceived ease of use, attitude toward use, and behavioral intention (5). In the present study, TAM is used as a measurement foundation, while JD-R provides the occupational health logic for examining how AI acceptance may be connected to teacher resources and health-related outcomes.

This distinction is especially important in intercultural teaching. Compared with general teaching contexts, intercultural or internationalized teaching involves language differences, cultural interpretation, international student support, classroom interaction norms, and cross-border collaboration practices. Teachers may accept AI for routine administrative or informational tasks, but still hesitate to use it in culturally sensitive teaching activities, such as explaining intercultural misunderstandings or designing culturally comparative cases. A context-specific measure of AI acceptance for intercultural teaching is therefore needed if AI acceptance is to be examined meaningfully in relation to teacher resources and occupational-health indicators.

Relatively few studies have linked AI acceptance with teachers’ occupational health outcomes. For university teachers, higher AI acceptance may reflect a perception that AI is usable for teaching, which may be associated with stronger intercultural teaching self-efficacy and autonomous teaching motivation. These teacher resources, in turn, may be associated with higher occupational wellbeing and lower emotional exhaustion. Recent studies have suggested meaningful associations among AI, teacher wellbeing, work engagement, anxiety, and burnout-related experiences, but more evidence is needed on the resource-related associations linking AI-related perceptions with occupational health (6–18).

Accordingly, this study adopted a two-sample cross-sectional design to examine university teachers’ occupational wellbeing and emotional exhaustion in the context of AI-supported intercultural teaching. The primary contribution was the context-specific development and preliminary measurement evaluation of the AI acceptance scale; the occupational-health analyses were exploratory associations examining whether AI acceptance was related to teacher-resource variables and health-related outcomes. Sample A was used to explore the structure of the AI acceptance scale, while Sample B was used to test measurement properties and examine associations with intercultural teaching self-efficacy, autonomous teaching motivation, occupational wellbeing, and emotional exhaustion.

## 2 Occupational health framework and hypotheses

### 2.1 AI acceptance as a work-related psychological orientation

TAM is one of the most widely used frameworks for explaining individual technology acceptance. Its core components include perceived usefulness, perceived ease of use, attitude toward use, and behavioral intention (5). In higher education, whether teachers accept AI tools depends not only on whether the tools are technically advanced, but also on whether teachers believe that the tools can help them complete teaching tasks, are easy to learn and operate, and are worth using in future teaching. We retain these four facets conceptually because they represent related but distinct stages of appraisal and willingness: perceived usefulness concerns expected task value, perceived ease of use concerns expected effort, attitude captures an evaluative stance, and behavioral intention captures readiness to use AI. Their conceptual distinction remains relevant even when empirical correlations are high, for three reasons. First, the facets served as a content-sampling frame: writing items for each facet ensured that the item pool covered expected value, expected effort, evaluation, and readiness to use AI rather than only one of these aspects. Second, TAM treats the facets as a sequence of related appraisals (beliefs, evaluation, and intention), so strong empirical convergence among them is consistent with a general acceptance factor that summarizes the facets; it does not by itself mean that the facets are absent as content. Third, retaining the facets conceptually allows future studies to test whether they become separable with refined items, larger samples, or alternative specifications such as second-order or bifactor models. Accordingly, the facets define the content of the AITA, whereas the overall score is the unit of analysis supported by the present data. In the present JD-R and self-determination perspective, favorable usefulness and ease appraisals may make AI-supported teaching feel worthwhile and manageable, which may be associated with autonomous interest or value identification; this association is the rationale for H2, but the cross-sectional design does not establish a motivational mechanism.

At the same time, teachers’ AI acceptance may be relevant to the JD-R model, but it should not be equated with an objectively available job resource. In this study, AI acceptance refers to teachers’ perceived usefulness, ease of use, attitude, and intention regarding AI in intercultural teaching. It is distinct from actual access to AI tools, frequency of use, and effective pedagogical application. Within a JD-R perspective, this appraisal may become resource-like when teachers have sufficient access, competence, and institutional support.

### 2.2 AI acceptance in intercultural teaching work

Existing studies on AI acceptance have often used general technology adoption frameworks to examine whether teachers or students are willing to use AI, whether they perceive it as useful, how institutional support influences adoption intention, and how teachers respond affectively and cognitively to ChatGPT and related generative AI tools (11, 12, 14, 19–23). These studies provide useful foundations, but they pay less attention to differences across specific teaching domains. Intercultural teaching is not simply an extension of ordinary classroom teaching. It often involves language transfer, cultural explanation, communication with international students, cross-border collaboration, and the handling of value differences.

In this study, AI acceptance for intercultural teaching is defined as university teachers’ context-specific appraisal of the usefulness, ease of use, attitude, and behavioral intention related to AI tools in intercultural or internationalized teaching tasks. The construct does not indicate that teachers actually use AI effectively or that AI is already available as a work resource. Rather, it captures a perceived readiness and usability appraisal that may be associated with teacher resources under supportive conditions. We conceptualize AI acceptance as a work-related psychological orientation: it reflects an appraisal of usefulness, ease of use, attitude, and intention, not actual access or use. It is distinct from autonomous teaching motivation, which concerns reasons and values for engaging in teaching, and from intercultural teaching self-efficacy, which concerns perceived capability to perform intercultural teaching tasks.

### 2.3 Teacher resource pathways: self-efficacy and autonomous motivation

Intercultural teaching self-efficacy refers to teachers’ belief in their ability to effectively complete intercultural or internationalized teaching tasks. It differs from general teaching self-efficacy because intercultural teaching requires teachers to address different language levels, cultural backgrounds, classroom participation norms, and forms of international collaboration. Teachers must not only organize teaching content, but also explain cultural differences, manage communication barriers, and design learning activities that promote cultural comparison and intercultural reflection (24–28).

In AI-supported teaching environments, teachers’ acceptance of AI may be associated with their intercultural teaching self-efficacy. When teachers perceive AI tools as useful for intercultural teaching and feel able to master these tools, they may be more likely to regard AI as an available teaching resource, which may be associated with greater confidence in handling complex intercultural teaching tasks.

Autonomous teaching motivation is another important resource for understanding teachers’ occupational experiences. In this study, it refers to teachers’ autonomous motivation to engage in intercultural or internationalized teaching, including interest, meaning, professional value identification, and willingness to improve teaching practice. According to self-determination theory, teachers’ experience of autonomy and meaning in work is closely related to sustained engagement and professional adaptation (29, 30). When teachers appraise AI as useful and manageable for intercultural tasks, these demanding tasks may be experienced as more feasible and personally worthwhile; within self-determination theory, perceived competence and the perceived value of an activity are conditions associated with intrinsic and identified regulation (29). This is the rationale for expecting AI acceptance to be positively associated with autonomous teaching motivation (H2), although the cross-sectional data cannot establish this motivational process.

### 2.4 Occupational wellbeing and emotional exhaustion as health outcomes

Teachers’ occupational wellbeing refers to the positive psychological state experienced in professional life, including professional identity, meaning, satisfaction, accomplishment, and positive affect. Occupational wellbeing reflects not only teachers’ individual condition, but also teaching quality, professional development, and organizational stability in education (1, 31, 32).

In contrast, emotional exhaustion is a central component of teacher burnout. It refers to the depletion of emotional energy caused by sustained teaching, communication, and management tasks. Occupational health research has shown that teachers are more likely to experience burnout and exhaustion when job demands are high and resources are insufficient (2, 3, 33, 34).

Based on the JD-R framework, this study examined whether AI acceptance was associated with teacher-resource variables and occupational-health indicators. A resource-pathway interpretation is theoretically plausible, but the cross-sectional design does not establish that AI acceptance produces self-efficacy, autonomous motivation, or improved occupational health.

### 2.5 Institutional support as an organizational resource

Perceived institutional support refers to the extent to which teachers feel that their university provides resources, training, technical services, policy recognition, and professional development support for their teaching work. Based on perceived organizational support theory, organizational support can be understood as an important job resource that helps teachers cope with work demands and maintain positive occupational states (35). In AI-supported intercultural teaching, institutional support is particularly important because teachers need not only operational skills, but also guidance on AI ethics, data security, course adaptation, and internationalized teaching norms.

Previous studies on AI adoption in education have shown that institutional support is associated with AI use intention, self-efficacy, and adoption behavior among teachers or students (9–12, 19, 21–23, 36–38). However, whether institutional support strengthens the association between AI acceptance and intercultural teaching self-efficacy remains uncertain. For this reason, the moderating role of perceived institutional support was tested as an exploratory hypothesis.

### 2.6 Hypotheses

*H1*: AI acceptance for intercultural teaching is positively associated with intercultural teaching self-efficacy.

*H2*: AI acceptance for intercultural teaching is positively associated with autonomous teaching motivation.

*H3*: Intercultural teaching self-efficacy and autonomous teaching motivation are positively associated with occupational well-being and negatively associated with emotional exhaustion.

*H4*: AI acceptance for intercultural teaching is indirectly associated with occupational well-being and emotional exhaustion through intercultural teaching self-efficacy and autonomous teaching motivation.

*H5, exploratory*: Perceived institutional support moderates the association between AI acceptance and intercultural teaching self-efficacy.

*H6, exploratory*: The indirect association through intercultural teaching self-efficacy varies by perceived institutional support.

## 3 Methods

### 3.1 Study design

This study adopted a cross-sectional questionnaire design to develop and preliminarily validate the AI Acceptance for Intercultural Teaching Scale, and to examine its associations with university teachers’ occupational wellbeing, emotional exhaustion, and related teacher resource variables. The study was conducted in two stages. In the first stage, Sample A was used for exploratory factor analysis to examine the latent structure of the AI acceptance scale. In the second stage, an independent Sample B was used for confirmatory measurement analysis, reliability and validity testing, structural association analysis, and exploratory moderation analysis. The two samples were collected in separate, non-overlapping time windows (Section 3.2), which helped reduce the risk of circular validation when developing and testing the scale; because responses were anonymous, the absence of individual-level overlap could not be verified directly.

Because the study was cross-sectional, all paths were interpreted as associations among variables, or as model-based indirect associations. No causal or temporal claims were made. The hypothesized framework is shown in Figure 1. Drawing on TAM and the JD-R model, the model treats AI acceptance as a technology acceptance variable situated in intercultural teaching, intercultural teaching self-efficacy and autonomous teaching motivation as teacher resource variables, and occupational wellbeing and emotional exhaustion as the positive and strain-related dimensions of teachers’ occupational health.

**Figure 1:**
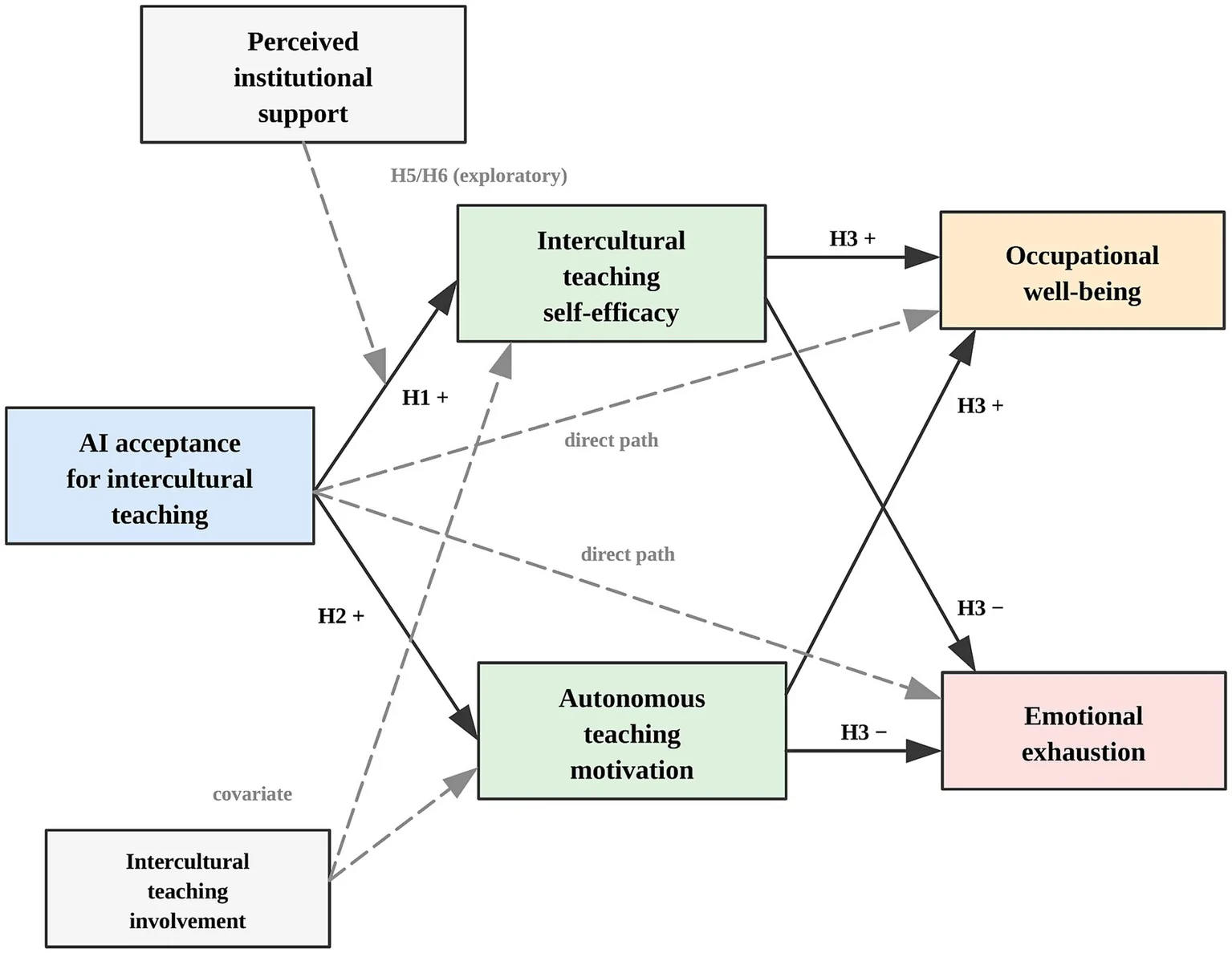
Hypothesized model. Solid lines summarize the hypothesized resource and outcome pathways corresponding to H1–H3, and H4 refers to the indirect associations formed by these paths; dashed lines denote the exploratory moderation path (H5–H6), the ITI covariate paths, and the direct paths from AITA to the outcomes, which were estimated without a separate hypothesis. For readability, the PIS main-effect path to ITSE and the ITI adjustment paths entering the OWB and EE equations are not drawn; their inclusion is specified in Methods and Table 7. AITA, AI acceptance for intercultural teaching; ITSE, intercultural teaching self-efficacy; ATM, autonomous teaching motivation; OWB, occupational wellbeing; EE, emotional exhaustion; PIS, perceived institutional support; ITI, intercultural teaching involvement. The study used a cross-sectional design; therefore, all paths represent theoretical associations rather than causal effects.

### 3.2 Participants and sampling

Participants were full-time university teachers in China. Eligible participants were required to have undertaken actual teaching duties during the most recent academic year and to pass the screening questions in the questionnaire. They were not required to have extensive prior intercultural-teaching experience. Intercultural teaching involvement was measured continuously with four frequency items covering internationalized courses, international students, COIL or virtual exchange, intercultural course content, and collaboration with overseas partners. In Sample B, the overall ITI score was *M* = 2.774 (SD = 0.809) on a 1–5 frequency scale. The four ITI items had means of 3.065 (SD = 1.191; *n* = 492), 2.655 (SD = 1.199; *n* = 493), 2.690 (SD = 1.170; *n* = 493), and 2.688 (SD = 1.205; *n* = 493), respectively. The descriptive sample profile also showed that 340 participants (68.687%) reported that their institution had a Sino-foreign cooperation, international cooperation, or international-student education program; 101 (20.404%) reported no such program and 54 (10.909%) were unsure. Institution types were teaching-and-research, teaching, research, and other institutions (*n* = 210, 148, 115, and 22, respectively). The prior international/intercultural-experience frequency field was reported as None, Once, 2–3 times, and 4 + times (None: *n* = 162); past-year AI-use frequency was recorded as Never, Occasionally, Monthly, Weekly, or Almost daily; and AI-related training/experience was recorded as a five-level ordinal indicator (Low, Mid-low, Medium, Mid-high, and High), treated as ordered levels rather than as a count of training sessions. The analysis dataset did not contain a geographic-region field, so regional representativeness could not be assessed. The distributions are summarized in Table 1.

**Table 1:** Demographic characteristics of Sample B.

Sample B demographics			
Variable	Level	*n*	Percent
Gender	Female	262	52.929
Gender	Male	231	46.667
Gender	Prefer not to say	2	0.404
Age group	31–40	206	41.616
Age group	41–50	146	29.495
Age group	≥51	72	14.545
Age group	≤30	71	14.343
Years of teaching	11–20	169	34.141
Years of teaching	6–10	139	28.081
Years of teaching	≤5	96	19.394
Years of teaching	≥21	91	18.384
Academic discipline	Social sciences	151	30.505
Academic discipline	STEM	135	27.273
Academic discipline	Humanities	92	18.586
Academic discipline	Medicine	65	13.131
Academic discipline	Arts	30	6.061
Academic discipline	Other	22	4.444
Institution type	Teaching and research	210	42.424
Institution type	Teaching	148	29.899
Institution type	Research	115	23.232
Institution type	Other	22	4.444
International-program availability	Yes	340	68.687
International-program availability	No	101	20.404
International-program availability	Unsure	54	10.909
Prior international/intercultural experience	None	162	32.727
Prior international/intercultural experience	2–3 times	141	28.485
Prior international/intercultural experience	Once	135	27.273
Prior international/intercultural experience	4+ times	57	11.515
Past-year AI-use frequency	Weekly	159	32.121
Past-year AI-use frequency	Monthly	123	24.848
Past-year AI-use frequency	Occasionally	120	24.242
Past-year AI-use frequency	Almost daily	59	11.919
Past-year AI-use frequency	Never	34	6.869
AI-training/experience level	Medium	185	37.374
AI-training/experience level	Mid-high	136	27.475
AI-training/experience level	Mid-low	83	16.768
AI-training/experience level	Low	49	9.899
AI-training/experience level	High	42	8.485

The raw Sample A included 380 submitted responses. After excluding 80 invalid responses, 300 valid cases remained after quality-control screening. Among these, 271 cases had complete responses on all 12 AI acceptance items and were therefore used in the complete-case EFA. Sample A included 27 of 300 participants who reported never using AI; the four intercultural-teaching-involvement items had means of 2.853, 2.669, 2.632, and 2.819 on the 1–5 frequency scale. Participants with limited prior exposure may have interpreted these contextual items as indicators of perceived involvement or readiness in the stated intercultural-teaching context; this interpretation is tentative and is treated as a limitation. The raw Sample B included 620 submitted responses. After excluding 125 invalid responses, 495 valid cases remained for confirmatory measurement analysis, reliability and validity testing, and structural association analysis. Because the survey was distributed through multiple forwarding channels and the invitation denominator was not recorded in the raw data package, a formal response rate could not be calculated. Exclusion criteria included failing the target-sample screening question, failing the attention-check items, completing the questionnaire in less than 180 s, and showing excessive straight-line responding across core items. Sample A included 152 women, 142 men, and 6 participants who preferred not to say; institution types were teaching-and-research (*n* = 126), teaching (*n* = 93), research (*n* = 68), and other (*n* = 13). In Sample A, 196 participants reported an institutional international/cooperation program, 68 reported none, and 36 were unsure. Geographic region was not recorded for either sample. Based on the recorded response timestamps, Sample A data were collected from 12 January to 18 February 2026 and Sample B data from 3 March to 18 April 2026.

### 3.3 Scale development procedure

The core instrument developed in this study was the AI Acceptance for Intercultural Teaching Scale. Scale development followed a theory construction, expert review, exploratory analysis, confirmatory analysis, and reliability and validity testing procedure. First, the initial items were generated based on the four-dimensional structure of TAM, including perceived usefulness, perceived ease of use, attitude toward use, and behavioral intention. To improve contextual relevance, all items were embedded in intercultural or internationalized teaching scenarios, such as intercultural case design, multilingual communication, support for international students, intercultural classroom interaction, and AI-assisted teaching tasks.

Second, the initial questionnaire was reviewed for content validity by experts. The expert review focused on the fit between items and constructs, the clarity of the intercultural teaching context, the readability of item wording, and the potential overlap between constructs. Expert review and content validity procedures were conducted with reference to established CVI recommendations (39, 40). Because the available expert review files record qualitative revision decisions but do not provide a complete auditable CVI scoring matrix for all retained items, this manuscript reports expert review as a content-refinement procedure and does not claim numerical CVI evidence.

Third, Sample A was used for exploratory factor analysis to inspect the empirical behavior of the 12 AI acceptance items. Maximum-likelihood extraction and direct oblimin rotation with Kaiser normalization were used because the dimensions were theoretically expected to be correlated. The EFA was treated as a diagnostic step rather than as definitive evidence for the final dimensional structure. Finally, Sample B was used for confirmatory analysis and reliability and validity testing, including confirmatory factor analysis, Cronbach’s alpha, composite reliability, average variance extracted, and HTMT discriminant validity assessment (41).

### 3.4 Measures

The questionnaire included screening and demographic information, intercultural teaching involvement, AI acceptance for intercultural teaching, intercultural teaching self-efficacy, autonomous teaching motivation, occupational wellbeing, emotional exhaustion, perceived institutional support, and a marker variable for common method bias. Unless otherwise specified, the core constructs were measured using a 5-point Likert scale ranging from 1 = strongly disagree to 5 = strongly agree. Intercultural teaching involvement and emotional exhaustion were measured as frequency items. The contextual background variables were used as descriptive ordinal indicators; the training/experience variable was not interpreted as a precise count of training sessions. The English item wording, response anchors, scoring rules, and construct-specific source/adaptation record are provided in Supplementary Tables S3, S4, and the bilingual administered questionnaire, including its Appendix A source table, is supplied as supplementary material; the construct composition and source summary are reported in Table 2. ITSE represents capability beliefs; ATM represents intrinsic or identified reasons for engaging in teaching; OWB represents occupational meaning, achievement, positive affect, and satisfaction; EE represents exhaustion symptoms; and PIS represents institutional resources and support. At the item level, ATM and OWB were separated by referent and item form: all ATM items completed the stem “I engage in intercultural/internationalized teaching because…” and therefore describe reasons for engaging in a specific teaching domain, whereas the OWB items refer to the teaching profession and work life as a whole and describe experienced states, such as accomplishment, satisfaction, and positive emotions. ITSE items, in turn, are phrased as capability statements (for example, “I can…” or “I am confident that…”). Consistent with this distinction, the HTMT values among ITSE, ATM, and OWB were 0.354–0.357 (Table 3). Related cross-national measurement work has also emphasized culturally responsive teacher self-efficacy in multicultural classrooms (27), but it was not used as an item source in the present adaptation.

**Table 2:** Scale composition and sources.

Scale composition					
Construct	Meaning	Items	Number of items	Source	Role in the paper
AITA	AI acceptance for intercultural teaching	D1–D12	12	Self-developed from TAM; PU/PEOU/ATT/BI contextualized to intercultural teaching	Main scale
PU	Perceived usefulness	D1–D3	3	AITA subdimension	Subdimension; reliability weaker
PEOU	Perceived ease of use	D4–D6	3	AITA subdimension	Subdimension; reliability weaker
ATT	Attitude	D7–D9	3	AITA subdimension	Subdimension; reliability weaker
BI	Behavioral intention	D10–D12	3	AITA subdimension	Subdimension; reliability weaker
ITSE	Intercultural teaching self-efficacy	E1–E6	6	Contextualized adaptation based on (24, 25); internationalized/cross-border teaching	Teacher-resource variable
ATM	Teaching motivation	F1–F6	6	Adapted from WTMST and SDT (29, 30); intrinsic and identified regulation	Teacher-resource variable
OWB	Occupational wellbeing	G1–G6	6	Adapted from (42, 43); meaning, achievement, positive affect, satisfaction	Positive occupational health outcome
EE	Emotional exhaustion	H1–H5	5	Contextualized emotional-exhaustion short form based on (44, 45); items were contextualized for this study; the original item wording was not reproduced	Negative occupational health outcome
PIS	Perceived institutional support	I1–I8	8	Contextualized POS/SPOS adaptation (46); AI-supported intercultural/international teaching support	Resource/moderator
ITI	Intercultural teaching involvement	C1–C4	4	Self-developed four-item frequency/breadth indicator	Covariate
MV	Marker variable	J1–J4	4	Self-developed neutral work-habit marker for common-method-bias diagnostics	Not a core construct

**Table 3:** Discriminant validity: HTMT results.

HTMT discriminant validity									
Construct	PU	PEOU	ATT	BI	ITSE	ATM	OWB	EE	PIS
PU	1	0.962	1.019	1.006	0.294	0.348	0.179	0.159	0.263
PEOU	0.962	1	1.037	0.967	0.253	0.278	0.120	0.175	0.208
ATT	1.019	1.037	1	1.006	0.274	0.315	0.163	0.165	0.285
BI	1.006	0.967	1.006	1	0.382	0.309	0.207	0.151	0.234
ITSE	0.294	0.253	0.274	0.382	1	0.354	0.357	0.230	0.281
ATM	0.348	0.278	0.315	0.309	0.354	1	0.355	0.165	0.356
OWB	0.179	0.120	0.163	0.207	0.357	0.355	1	0.323	0.356
EE	0.159	0.175	0.165	0.151	0.230	0.165	0.323	1	0.291
PIS	0.263	0.208	0.285	0.234	0.281	0.356	0.356	0.291	1

AI acceptance for intercultural teaching was the core scale developed in this study. It included 12 items initially organized into four theory-based content facets: perceived usefulness, perceived ease of use, attitude toward use, and behavioral intention, with three items for each facet. Because the empirical results did not clearly distinguish the four facets, the overall AITA score was used as the primary measure in structural analyses; the facet scores were not treated as fully validated independent subscales. Perceived usefulness and perceived ease of use represent instrumental appraisals, attitude represents evaluative acceptance, and behavioral intention represents willingness or readiness to use AI; the current data nevertheless show substantial empirical convergence among these facets.

Intercultural teaching self-efficacy was measured with six items assessing confidence in teaching effectively in international or cross-border settings, adapting teaching to students from different cultural backgrounds, handling cultural differences and misunderstandings, designing intercultural learning activities, and communicating with international students or overseas partners (24, 25). Autonomous teaching motivation was measured with six items focusing on intrinsic enjoyment, personal importance, professional values, and identification with intercultural or internationalized teaching (29, 30). Occupational wellbeing was measured with six items covering accomplishment, professional identity, job satisfaction, meaning, positive affect, and work-related wellbeing (42, 43). Emotional exhaustion was measured with five frequency items as a contextualized short measure informed by the burnout literature (44, 45); it was not treated as a licensed reproduction of a proprietary instrument.

Perceived institutional support was measured with eight items assessing whether teachers perceived their university as providing resources, training, technical support, ethical guidance, policy recognition, and professional development support for AI-assisted intercultural or internationalized teaching, drawing on perceived organizational support theory (46). The marker variable for common method bias consisted of four neutral work-habit items, following recommendations on common method bias control and assessment (47, 48). The marker variable was used as a diagnostic check rather than as a full latent marker-factor correction.

### 3.5 Control variables

The structural models included intercultural teaching involvement (ITI) as the prespecified covariate because it was directly tied to the study context and the opportunity to use AI in intercultural teaching. Demographic and professional background variables, including gender, age, years of teaching, discipline, institution type, AI-use experience, and training experience, were described in the sample profile but were not entered into the main structural models. This narrower covariate strategy was used to keep the model aligned with the associations examined in this study and to avoid overfitting given the number of latent constructs.

### 3.6 Data analysis strategy

Data analysis consisted of four parts: data cleaning, scale diagnostics, structural association testing, and sensitivity analysis. IBM SPSS Statistics 27.0.1 was used for EFA, Cronbach’s alpha, descriptive statistics, correlation analysis, and score-based regression analyses. The EFA used listwise complete cases, maximum-likelihood extraction, four factors fixed *a priori*, and direct oblimin rotation with Kaiser normalization; the rotation converged after 11 iterations. CFA and SEM were conducted in R 4.6.1 (lavaan 0.6.21, lavaan.mi 0.1.0, and mice 3.19.0) using lavaan with robust maximum likelihood (MLR) estimation. CR and AVE were calculated from the standardized loadings of the complete-case 11-factor CFA using the formulas of Fornell and Larcker (49), and discriminant validity was assessed with the heterotrait-monotrait ratio (HTMT), computed as the arithmetic-mean ratio of absolute pairwise item correlations so that values are positive for negatively related constructs such as EE (41); these calculations are reproduced in the Analysis Code Appendix. Scale scores were computed as the arithmetic mean of the available items of each scale, so that a score was missing only when all items of that scale were missing; because item-level missingness was low (at most 9.3% for any item; Supplementary Table S1), all 495 Sample B cases had non-missing scores on every construct used in the structural analyses. These non-imputed scale scores were used for the primary analyses. As a sensitivity analysis, item-level missing data were handled with mice multiple imputation (*m* = 5), and CFA/SEM model tests were pooled with lavaan.mi using the D2 rule. Indirect-association bootstrap intervals were treated as sensitivity summaries rather than formal D2-pooled bootstrap tests. The score-based bootstrap procedure was run in R and used 5,000 resamples with seed 20,260,701. Across the 51 item columns used in measurement analyses, the complete-case sample was *n* = 242 before imputation; the imputed sensitivity analyses retained 495 cases.

The CFA, composite reliability (CR), average variance extracted (AVE), HTMT, and latent model-fit analyses were conducted in R as described above. To examine whether the AITA facets were empirically distinguishable, we also compared an AITA-only one-factor CFA with the correlated four-factor AITA specification in cleaned Sample B (*n* = 495), using robust maximum likelihood with full-information maximum likelihood for item-level missingness. The 11-factor measurement CFA, including the four AITA facets and the remaining constructs, was retained as a theory-specified diagnostic model; this did not establish that the four AITA facets were empirically distinct. Because the four AITA facets overlapped strongly (high HTMT, modest CR/AVE) and a one-factor model fitted as well, the overall AITA score was used for the primary structural analyses.

The primary structural association analyses used observed scale scores and regression models to examine associations of overall AI acceptance with intercultural teaching self-efficacy, autonomous teaching motivation, occupational wellbeing, and emotional exhaustion. Intercultural teaching involvement was included as a covariate where specified. The latent CFA and SEM were treated as sensitivity analyses for measurement and overall model fit only. The CFA used 51 items including the four marker items (J1–J4), whereas the latent SEM used 47 substantive items (C1–I8) and excluded the marker items; the latter specified one overall AITA factor together with ITSE, ATM, OWB, EE, PIS, and ITI. In the latent SEM, the residual covariances between ITSE and ATM and between OWB and EE were fixed to zero. Because the OWB–EE constraint is restrictive (in the score-based models, the two outcomes remained correlated after the predictors were taken into account), the latent SEM was used only as a check of global model fit, and its path estimates were not used to test the hypotheses. Indirect associations were estimated as products of standardized regression coefficients, with percentile intervals from 5,000 bootstrap resamples (seed 20,260,701) in the score-based workflow and are reported as model-based indirect associations, not causal mediation effects. The bootstrap summaries were not formal D2-pooled bootstrap confidence intervals. The non-imputed and multiple-imputation score-based regressions (pooled with Rubin’s rules) were compared for direction and substantive interpretation (Supplementary Table S6).

### 3.7 Ethics statement

This study used an anonymous questionnaire survey and did not collect directly identifiable personal information, such as names, phone numbers, or identification numbers. The first page of the questionnaire explained the study purpose, voluntary participation, data use, anonymity, and the right to withdraw. All participants were required to read the electronic informed consent statement and confirm their voluntary participation before answering the survey. The data were used only for academic research and aggregate statistical analysis and were stored in a de-identified format. The study was reviewed and approved by the Universiti Malaya Research Ethics Committee (UMREC), Universiti Malaya (reference no. UM.TNC2/UMREC_4040; approved December 2024, valid until December 2027). The participants provided their electronic informed consent to participate in this study.

## 4 Results

### 4.1 Data cleaning and preliminary analysis

The raw Sample A contained 380 responses. Based on the target-sample screening question, attention checks, response time, and straight-line responding rules, 80 invalid responses were excluded. The final Sample A included 300 valid responses and was used for exploratory factor analysis. The raw Sample B contained 620 responses. After excluding 125 invalid responses, 495 valid responses remained for confirmatory analysis, reliability and validity testing, and structural association analysis. After cleaning, both datasets met the prespecified quality-control criteria.

Descriptive statistics and correlations among the main constructs in Sample B are presented in Table 4. The absolute values of skewness were below 1, and kurtosis values were also within an acceptable range, suggesting that the main construct scores did not show serious non-normality. AI acceptance was positively correlated with intercultural teaching self-efficacy, autonomous teaching motivation, and perceived institutional support. Intercultural teaching self-efficacy and autonomous teaching motivation were positively correlated with occupational wellbeing and negatively correlated with emotional exhaustion. Occupational wellbeing was negatively correlated with emotional exhaustion.

**Table 4:** Descriptive statistics and correlations among main constructs.

Descriptive statistics						
Construct	Mean	std	Min	Max	Skew	Kurt
AITA	3.593	0.673	1.500	5	−0.274	−0.342
ITSE	3.391	0.800	1.167	5	−0.190	−0.535
ATM	3.671	0.689	1.500	5	−0.325	−0.357
OWB	3.548	0.767	1.167	5	−0.248	−0.643
EE	2.405	0.727	1	4.400	0.308	−0.407
PIS	2.924	0.764	1.125	4.875	0.100	−0.432
ITI	2.774	0.809	1	5	0.343	−0.264
MV	3.050	0.709	1	4.750	−0.169	−0.305

Correlations								
Construct	AITA	ITSE	ATM	OWB	EE	PIS	ITI	MV
AITA	1	0.256	0.193	0.126	−0.114	0.225	0.141	−0.012
ITSE	0.256	1	0.270	0.320	−0.199	0.231	0.211	−0.027
ATM	0.193	0.270	1	0.273	−0.138	0.248	0.122	0.028
OWB	0.126	0.320	0.273	1	−0.268	0.276	0.074	0.035
EE	−0.114	−0.199	−0.138	−0.268	1	−0.229	−0.094	−0.046
PIS	0.225	0.231	0.248	0.276	−0.229	1	0.187	−0.038
ITI	0.141	0.211	0.122	0.074	−0.094	0.187	1	−0.030
MV	−0.012	−0.027	0.028	0.035	−0.046	−0.038	−0.030	1

Regarding common method bias, Harman’s single-factor test (one unrotated factor extracted by maximum likelihood from the 51 items; listwise complete cases, *n* = 242) showed that this factor explained 12.48% of the total variance, and the marker variable showed near-zero correlations with the focal constructs; its internal consistency was limited (alpha = 0.438). These diagnostics did not suggest an overwhelming single-factor pattern, but neither procedure can rule out common method bias. Because the study relied on same-source, cross-sectional self-report data, the observed associations were interpreted cautiously.

### 4.2 Exploratory factor analysis in Sample A

Sample A was used to examine the latent structure of the AI Acceptance for Intercultural Teaching Scale. Because the scale was designed according to the four-dimensional structure of TAM, four factors were extracted using maximum-likelihood estimation with direct oblimin rotation and Kaiser normalization. Although 300 cases remained after quality-control cleaning, 271 cases had complete responses on all 12 AI acceptance items and were included in the complete-case EFA. The 29 excluded cases reflected item-level nonresponse on the core AITA items rather than failures of the prespecified quality-control rules. The KMO value was 0.940, and Bartlett’s test of sphericity was significant, chi-square(66) = 1,038.680, *p* < 0.001, indicating that the data were suitable for factor analysis.

The EFA results are shown in Table 5. The table reproduces the SPSS Pattern Matrix, with loadings below |0.30| suppressed. The rotated pattern did not cleanly reproduce the theorized four-dimensional structure: most items loaded on Factor 1, D3 showed a near-unit negative loading on Factor 2, D11 had a cross-loading pattern, and D2, D4, and D7 loaded on Factor 4 rather than forming the proposed TAM subdimensions. Because factor signs are indeterminate under rotation, the signs are not interpreted substantively. The EFA therefore provides evidence of factorability and item-pool instability, rather than strong evidence for four stable subscales.

**Table 5:** Exploratory factor analysis of AI acceptance items in Sample A.

EFA adequacy				
Sample	*n*	KMO	Bartlett *χ*^2^	Bartlett *p*
A	271	0.940	1038.680	<0.001

EFA pattern matrix				
Item	Factor 1	Factor 2	Factor 3	Factor 4
D1	0.673			
D2				−0.389
D3		−0.986		
D4				−0.700
D5	0.619			
D6	0.470			
D7				−0.425
D8	0.542			
D9	0.570			
D10	0.623			
D11	0.338		−0.465	
D12	0.576			

Maximum-likelihood extraction with four factors fixed a priori and direct oblimin rotation with Kaiser normalization; listwise complete cases, *n* = 271. Loadings below |0.30| are suppressed. The SPSS output flagged a communality estimate greater than 1, so the EFA is interpreted as a diagnostic analysis rather than definitive evidence of four independent dimensions.

### 4.3 Confirmatory measurement model, reliability, and validity

The pooled 11-factor measurement CFA showed acceptable global fit in the sensitivity analysis (chi-square/df = 1.117, CFI = 0.973, TLI = 0.971, RMSEA = 0.015; Supplementary Table S2). The pooled CFA uses 51 observed items (C1–C4, D1–D12, E1–E6, F1–F6, G1–G6, H1–H5, I1–I8, and J1–J4) and 11 correlated factors. The latent SEM uses the 47 substantive items C1–I8, excludes the four marker items J1–J4, specifies one overall AITA factor plus ITSE, ATM, OWB, EE, PIS, and ITI, and estimates the prespecified structural paths. Thus df = 1,169 for the 51-item CFA and df = 1,018 for the different 47-item SEM are not interpreted as a simple path-addition comparison. The complete-case 11-factor measurement CFA was also broadly acceptable (chi-square/df = 1.166, CFI = 0.929, TLI = 0.922, RMSEA = 0.026). An AITA-only model comparison yielded similarly close fit for the one-factor model, chi-square(54) = 49.70, CFI = 1.000, TLI = 1.004, RMSEA = 0.000, SRMR = 0.023, and the correlated four-factor model, chi-square(48) = 45.49, CFI = 1.000, TLI = 1.003, RMSEA = 0.000, SRMR = 0.022. The small fit difference does not establish that the four AITA facets are empirically distinct (Supplementary Table S5). These fit indices do not by themselves establish strong convergent or discriminant validity for every AITA subdimension; the low subdimension AVE values and high HTMT values were therefore treated as substantive limitations.

Reliability and convergent validity results are reported in Table 6. The overall AITA scale showed good internal consistency, with a Cronbach’s alpha of 0.858, but this coefficient does not establish unidimensionality. All reported core constructs had AVE below 0.50: ITSE = 0.401, ATM = 0.307, OWB = 0.348, EE = 0.351, and PIS = 0.374; the AITA subdimensions also had AVE values between 0.310 and 0.374. ITI showed alpha = 0.611 and CR = 0.644, while the marker variable showed alpha = 0.438 and CR = 0.424. These results indicate that the evidence is preliminary and that the overall AITA score is used pragmatically as a composite summary rather than as proof of a unidimensional scale. For ITSE, ATM, OWB, EE, and PIS, CR values (0.721–0.826) indicated acceptable composite reliability, but AVE values below 0.50 mean that more than half of the item variance was not explained by the intended constructs. This modest convergent validity implies that the observed scale scores contain substantial measurement error, which is likely to attenuate the score-based associations reported below; it is therefore treated as a limitation rather than set aside.

**Table 6:** Primary reliability and convergent validity estimates.

Reliability and validity					
Construct	Items	Alpha	CR	AVE	Standardized loading range
ITI	4	0.611	0.644	0.313	0.496–0.640
PU	3	0.658	0.641	0.374	0.551–0.651
PEOU	3	0.574	0.571	0.310	0.456–0.617
ATT	3	0.594	0.574	0.311	0.515–0.614
BI	3	0.592	0.577	0.315	0.497–0.617
AITA	12	0.858			
ITSE	6	0.787	0.800	0.401	0.543–0.706
ATM	6	0.732	0.721	0.307	0.335–0.614
OWB	6	0.780	0.759	0.348	0.461–0.671
EE	5	0.689	0.729	0.351	0.555–0.669
PIS	8	0.811	0.826	0.374	0.531–0.695
MV	4	0.438	0.424	0.165	0.278–0.555

The main table reports one primary set of estimates: SPSS-native Cronbach’s alpha, and composite reliability (CR), average variance extracted (AVE), and the standardized loading range from the complete-case 11-factor CFA (*n* = 242), with CR and AVE calculated using the Fornell–Larcker formulas. Imputation-specific estimates are not reported separately.

In Table 6, the AITA overall row reports the total-scale Cronbach’s alpha only; CR, AVE, and the loading range are left blank because Table 6 reports estimates from the complete-case 11-factor CFA, in which AITA is represented by its four facets rather than by a single factor. The AITA-only comparison (Supplementary Table S5) indicates that the 12 items are compatible with a single general factor, which is the basis for using the overall score. The main table presents one primary estimate set; imputation-specific reliability estimates are not reported separately.

The reliability and validity evidence should therefore be interpreted cautiously. The low AVE values across the core constructs, the weaker ITI reliability, and the low marker-variable alpha indicate that internal consistency and convergent validity are not uniformly strong. The main structural analyses used the overall AITA score as a pragmatic composite summary, while the subdimension-level findings were treated as preliminary; the data are compatible with a single general factor, but unidimensionality is not regarded as established.

Discriminant validity results are shown in Table 3. The external constructs showed generally interpretable empirical separation, but the HTMT results indicated substantial overlap among the four AITA facets, with several values approaching or exceeding 1.0. Thus, the four facets should not be interpreted as empirically distinct subscales in this sample. They are retained as theory-based content facets, while the overall AITA score is used as the primary measure.

### 4.4 Structural and indirect associations

The structural association model was used to examine associations among overall AI acceptance, teacher-resource variables, and occupational-health outcomes. The latent SEM showed acceptable global fit (Supplementary Table S2), but because it fixed the OWB–EE residual covariance to zero (Section 3.6), it was used only as a global-fit check; the hypothesis tests were based on observed scale-score regressions. AI acceptance was positively associated with intercultural teaching self-efficacy and autonomous teaching motivation. Self-efficacy was positively associated with occupational wellbeing and negatively associated with emotional exhaustion, whereas autonomous motivation was positively associated with occupational wellbeing but was not clearly associated with emotional exhaustion. The direct associations of AI acceptance with occupational wellbeing and emotional exhaustion were not significant after teacher-resource variables were included.

The primary path results are presented in Table 7 (standardized score-based regression paths; the multiple-imputation regressions in Supplementary Table S6 are a sensitivity check of these paths). AI acceptance was positively associated with intercultural teaching self-efficacy, beta = 0.200, *p* < 0.001, supporting H1. AI acceptance was also positively associated with autonomous teaching motivation, beta = 0.180, *p* < 0.001, supporting H2. Intercultural teaching self-efficacy was positively associated with occupational wellbeing, beta = 0.263, *p* < 0.001; autonomous teaching motivation was also positively associated with occupational wellbeing, beta = 0.199, *p* < 0.001. Intercultural teaching self-efficacy was negatively associated with emotional exhaustion, beta = −0.154, *p* = 0.001. The negative association between autonomous teaching motivation and emotional exhaustion was marginal, beta = −0.080, *p* = 0.083. These findings partially supported H3. The corresponding structural path diagram is shown in Figure 2.

**Table 7:** Structural paths and indirect associations.

Structural paths							
Outcome	Predictor	Beta	SE	*t*	*p*	*R* ^2^	sig
ITSE	AITA	0.200	0.044	4.576	<0.001	0.120	***
ITSE	PIS	0.154	0.045	3.454	<0.001	0.120	***
ITSE	AITA × PIS	0.027	0.045	0.601	0.548	0.120	ns
ITSE	ITI	0.153	0.043	3.539	<0.001	0.120	***
ATM	AITA	0.180	0.044	4.041	<0.001	0.046	***
ATM	ITI	0.096	0.044	2.169	0.031	0.046	*
OWB	AITA	0.022	0.044	0.499	0.618	0.141	ns
OWB	ITSE	0.263	0.045	5.813	<0.001	0.141	***
OWB	ATM	0.199	0.044	4.524	<0.001	0.141	***
OWB	ITI	−0.009	0.043	−0.201	0.841	0.141	ns
EE	AITA	−0.053	0.046	−1.153	0.249	0.052	ns
EE	ITSE	−0.154	0.047	−3.247	0.001	0.052	**
EE	ATM	−0.080	0.046	−1.737	0.083	0.052	ns
EE	ITI	−0.045	0.045	−0.985	0.325	0.052	ns

Effect	Estimate	Bootstrap SE	95% CI lower	95% CI upper	Supported by CI	*N*
Indirect association: AITA to ITSE to OWB	0.053	0.014	0.028	0.083	yes	495
Indirect association: AITA to ATM to OWB	0.036	0.012	0.016	0.061	yes	495
Indirect association: AITA to ITSE to EE	−0.031	0.012	−0.058	−0.009	yes	495
Indirect association: AITA to ATM to EE	−0.014	0.009	−0.035	0.001	no	495
Moderated indirect association: ITSE to OWB	0.007	0.011	−0.013	0.029	no	495
Moderated indirect association: ITSE to EE	−0.004	0.007	−0.019	0.008	no	495

Indirect associations are products of standardized observed-score regression coefficients. The 95% confidence intervals are percentile intervals from 5,000 row-resampling bootstrap replicates (bootstrap seed 20,260,701; *N* = 495 cases, all with non-missing available-item scale scores). The five-imputation sensitivity regressions (Supplementary Table S6) were pooled with Rubin’s rules, and the CFA/SEM model tests with the D2 rule; neither set of sensitivity estimates is combined with the bootstrap intervals in this table. *p < 0.05; **p < 0.01; ***p < 0.001; ns, not statistically significant (*p* ≥ 0.05).

**Figure 2:**
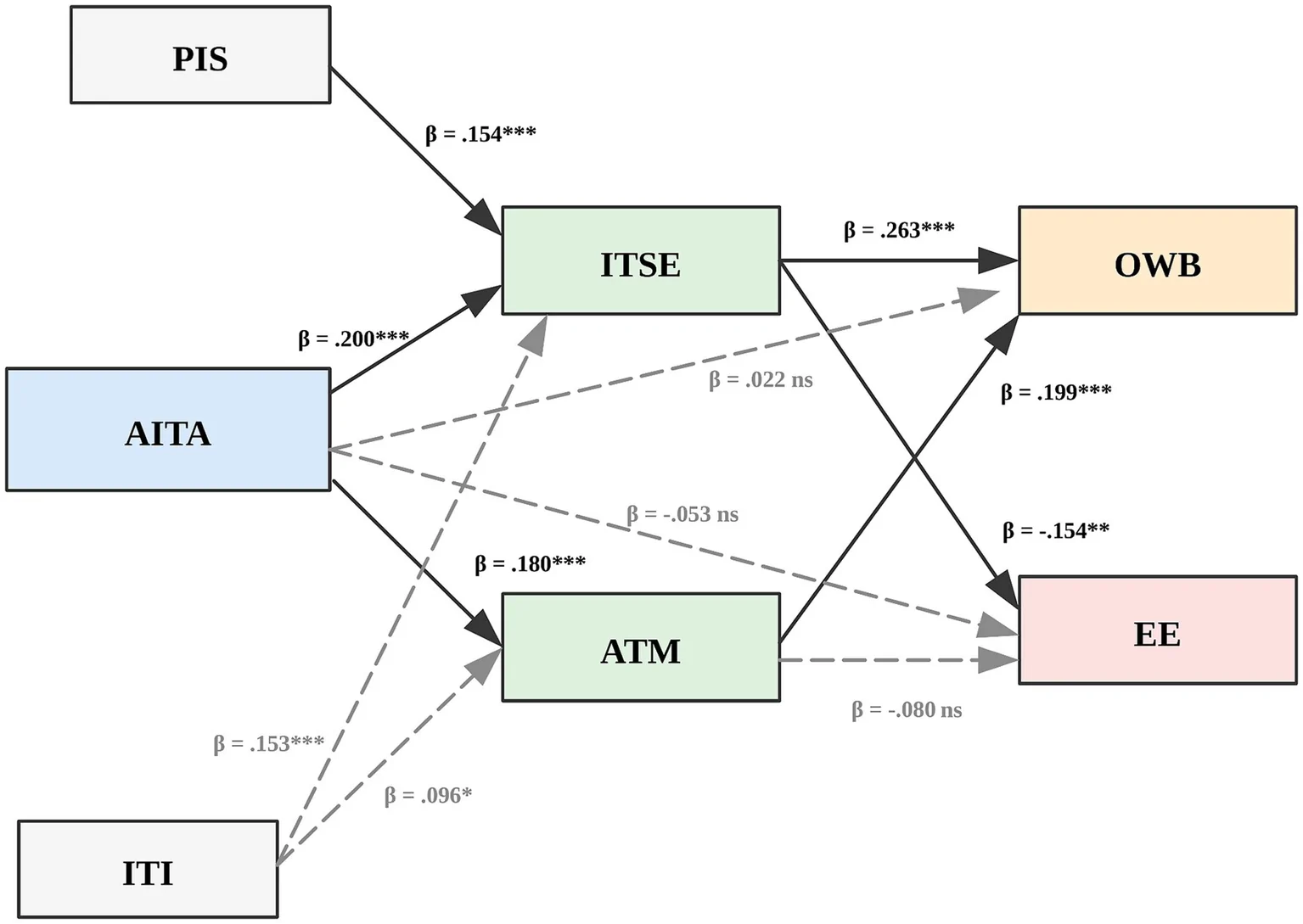
Structural path results. Coefficients are from the primary observed-score regression reported in Table 7 (*n* = 495). The path labels show the standardized coefficients used in Table 7; the graphic uses “*β* =” to label the standardized path coefficients. Solid arrows indicate statistically significant primary paths. Dashed grey arrows denote covariate paths and non-significant or marginal paths; ITI adjustment paths entering the OWB and EE equations are included in the regression models but omitted from the diagram for readability, and the direct AITA-to-outcome paths are non-significant. The AITA × PIS interaction term in the ITSE equation (beta = 0.027, *p* = 0.548; Table 7) is not drawn. The paths represent cross-sectional associations, not causal effects. *p < 0.05; **p < 0.01; ***p < 0.001; ns, not statistically significant (*p* ≥ 0.05).

Overall, the structural association results showed that AI acceptance was positively associated with intercultural teaching self-efficacy and autonomous teaching motivation. Intercultural teaching self-efficacy and autonomous teaching motivation were, in turn, positively associated with occupational wellbeing, while intercultural teaching self-efficacy was also significantly and negatively associated with emotional exhaustion. By contrast, after teacher resource variables were included in the model, the direct associations of AI acceptance with occupational wellbeing and emotional exhaustion were not significant.

The bootstrap sensitivity summaries were consistent with indirect associations from AI acceptance to occupational wellbeing through self-efficacy and autonomous motivation, and to emotional exhaustion through self-efficacy. The indirect association through autonomous motivation to emotional exhaustion was not supported. These results provide partial support for H4 at the level of model-based indirect associations, but they are cross-sectional and should not be interpreted as causal mediation or as formal D2-pooled bootstrap tests.

### 4.5 Exploratory moderation analysis

The exploratory moderation analysis showed that both AI acceptance and perceived institutional support were positively associated with intercultural teaching self-efficacy, but their interaction term was not significant. Thus, perceived institutional support did not significantly moderate the association between AI acceptance and intercultural teaching self-efficacy, and H5 was not supported. The indices of moderated indirect association were also not significant, meaning that H6 was not supported. The interaction term was non-significant in both specifications: beta = 0.027, *p* = 0.548 in the covariate-adjusted self-efficacy model in Table 7, and beta = 0.024, *p* = 0.588 in the mean-centered moderation model in Table 8. The small differences in the AI acceptance and perceived institutional support main-effect coefficients between the two tables (for example, beta = 0.200 versus beta = 0.216 for AI acceptance) reflect the inclusion of the ITI covariate in the Table 7 model, not conflicting findings.

**Table 8:** Exploratory moderation analysis.

Exploratory moderation				
Term	*B*	Beta	*p*	Interpretation
Mean-centered AITA	0.257	0.216	<0.001	Significant positive main association
Mean-centered PIS	0.188	0.179	<0.001	Significant positive main association
AITA × PIS	0.039	0.024	0.588	Not significant; keep as exploratory

This model regressed intercultural teaching self-efficacy on mean-centered AI acceptance (Mean-centered AITA), mean-centered perceived institutional support (Mean-centered PIS), and their interaction (AITA x PIS), estimated without the ITI covariate (*R*^2^ = 0.098). This centered model provided the basis for the simple-slope summary (Supplementary Figure S1). The corresponding covariate-adjusted estimates, which include ITI and use construct scale scores, are reported in the self-efficacy rows of Table 7; the two models differ in specification and are not competing estimates of the same coefficient. *B*, unstandardized coefficient; beta, standardized coefficient.

This finding suggests that perceived institutional support may itself be an organizational resource related to teacher self-efficacy, but the present data do not show that it changes the association between AI acceptance and self-efficacy. The moderation result should therefore be interpreted as exploratory and non-confirmatory.

### 4.6 Robustness and sensitivity analyses

To examine the stability of the findings, this study compared the primary non-imputed analyses with multiple-imputation analyses. After data cleaning, Sample B contained 495 valid responses, with low item-level missingness (at most 9.3% for any item; Supplementary Table S1). Because the missing responses were dispersed across cases, 253 of the 495 cases (51.1%) had at least one missing response among the 51 measurement items, and item-level complete-case analysis reduced the sample to 242 cases for the 51-item measurement model, whereas the available-item scale scores were non-missing for all 495 cases. After mice multiple imputation (*m* = 5) and lavaan.mi pooling using the D2 method, CFA and SEM used all 495 cases and showed acceptable fit. The complete-case CFA (*n* = 242) also showed acceptable fit, suggesting that the main measurement structure was consistent across different missing-data handling approaches.

For the structural paths, the multiple-imputation and non-imputed score-regression estimates were highly similar in magnitude and direction (Supplementary Table S6); no standardized coefficient differed by more than 0.004. The ITSE–EE path was −0.155 under multiple imputation and −0.154 in the non-imputed analysis, and both estimates were negative and statistically significant; the ATM–EE path remained marginal in both analyses (beta = −0.084, *p* = 0.069, and beta = −0.080, *p* = 0.083). AI acceptance was positively associated with intercultural teaching self-efficacy and autonomous teaching motivation; intercultural teaching self-efficacy and autonomous teaching motivation were positively associated with occupational wellbeing; and intercultural teaching self-efficacy was negatively associated with emotional exhaustion. The direct paths from AI acceptance to occupational wellbeing and emotional exhaustion remained non-significant after teacher resource variables were included. Exploratory moderation paths were not supported across the analyses.

Taken together, the sensitivity analyses suggest that the direction of the main score-based associations was broadly stable across non-imputed and imputed analyses. They do not establish that one missing-data method is definitively correct, and they do not convert the indirect associations into causal mediation evidence. The most consistent pattern was that AI acceptance was associated with teacher-resource variables, especially intercultural teaching self-efficacy, while the explained variance in the occupational-health outcomes remained modest.

## 5 Discussion

### 5.1 Main findings

This study developed a context-specific measure of AI acceptance for intercultural teaching and examined its cross-sectional associations with teacher resources and occupational-health indicators. The strongest contribution is the preliminary measurement work: the overall AITA score showed good internal consistency, whereas the four theory-based facets were not clearly distinguishable empirically. The structural findings provide modest association evidence rather than evidence that AI acceptance directly improves occupational health.

The structural association results are compatible with a resource-related pattern rather than a direct association between AI acceptance and the occupational-health outcomes. AI acceptance was associated with both self-efficacy and autonomous motivation. Self-efficacy was associated with higher occupational wellbeing and lower emotional exhaustion, whereas autonomous motivation was associated mainly with occupational wellbeing. Within the JD-R interpretation, self-efficacy represents an efficacy-related personal resource, whereas autonomous motivation represents an internal motivational resource. This distinction helps preserve the different content of the two resource pathways rather than treating all resources as interchangeable. The pattern is also consistent with the dual-process logic of the JD-R model (3, 4): resources are expected to relate mainly to the motivational process and its positive outcomes, such as wellbeing, whereas emotional exhaustion is expected to arise mainly from job demands through the health-impairment process. Autonomous motivation, as a motivational resource, was accordingly associated with occupational wellbeing but only marginally with exhaustion, whereas self-efficacy, which may also help teachers appraise demanding intercultural tasks as manageable, was associated with both outcomes. Because no formal coefficient-difference test was conducted, this interpretation concerns the pattern of significance rather than a demonstrated difference between the two resources. The score-based models explained *R*^2^ = 0.141 for occupational wellbeing and *R*^2^ = 0.052 for emotional exhaustion. The low explained variance in exhaustion is consistent with the same logic: the model contained resource variables but no measures of job demands, such as teaching workload, administrative pressure, or AI-related role demands, which JD-R identifies as the main antecedents of exhaustion. The present model therefore should not be read as an account of teachers’ exhaustion.

The exploratory moderation results did not support perceived institutional support as a boundary condition. Although perceived institutional support was positively associated with intercultural teaching self-efficacy, it did not significantly change the strength of the association between AI acceptance and self-efficacy. From an occupational health perspective, this pattern is compatible with institutional support operating as a general organizational resource rather than as a moderator of the association between AI acceptance and self-efficacy.

### 5.2 Relation to public mental health and occupational Health Research

The findings are broadly consistent with both TAM and the JD-R framework, but the two frameworks serve different roles. TAM provides the theoretical content for the AITA item pool, whereas JD-R provides a framework for interpreting why a perceived AI-usability appraisal might be related to teacher resources and occupational-health indicators. The present results do not show that AI acceptance itself is an objective job resource, nor that it causes changes in self-efficacy, motivation, or occupational health.

Compared with previous research on AI adoption in education, this study further highlights the importance of the teaching context. Intercultural teaching involves cultural explanation, multilingual communication, support for international students, and cross-border collaboration. These tasks differ from general classroom teaching, and recent work on AI-enabled intercultural learning and COIL further suggests that AI use may reshape intercultural learning opportunities and challenges (13, 16). Accordingly, teachers’ acceptance of AI cannot be fully captured by a general measure of technology adoption intention; it needs to be assessed in relation to specific intercultural teaching tasks.

From the perspective of teacher occupational health and public mental health, the findings reinforce the importance of resources rather than technology exposure alone. Recent research on AI, teacher wellbeing, work engagement, anxiety, and burnout has indicated that technological change may be connected to teachers’ work experiences (6–15, 17, 18). The present study adds that, in intercultural teaching contexts, the observed pattern is compatible with resource-related associations involving intercultural teaching self-efficacy and autonomous motivation, whereas the direct associations between AI acceptance and occupational-health outcomes were not significant. This pattern should not be interpreted as evidence of a tested mechanism.

### 5.3 Tentative implications for occupational health promotion in universities

First, the findings suggest several hypotheses for universities seeking to support teachers during AI-supported intercultural or internationalized teaching. Universities may consider scenario-based guidance, ethical and data-security support, technical assistance, and opportunities for peer exchange. However, these are proposals for future implementation and intervention research: the present study did not compare training formats, evaluate institutional policies, or estimate the occupational-health effects of specific interventions.

Second, future professional-development studies could examine whether training that supports intercultural teaching self-efficacy is more useful than training focused only on software functions. The present study did not compare training formats or evaluate training effects. Any occupational-health implications of such training therefore remain to be tested.

Third, although perceived institutional support did not significantly moderate the association between AI acceptance and self-efficacy, it was itself positively associated with self-efficacy. Future implementation studies could therefore evaluate whether concrete and visible forms of support, such as AI teaching ethics guidelines, data-security standards, technical support, intercultural teaching resources, incentives for teaching innovation, and opportunities for peer exchange, are followed by changes in teacher resources and occupational-health indicators. Whether such measures function as occupational health promotion, rather than only as digital transformation policies, remains a proposition to be tested.

### 5.4 Limitations and future research

This study has several limitations. First, the cross-sectional questionnaire design does not allow causal or temporal inferences. All paths in this article should therefore be interpreted as associations or model-based indirect associations. Future studies could use longitudinal designs, tracking data, or quasi-experimental methods to further examine the dynamic relationships among AI acceptance, teacher resources, and occupational health.

Second, although the study used anonymous responses, attention checks, a marker variable, and Harman’s single-factor test, these procedures cannot eliminate common method bias. All focal variables were collected from the same respondents at one time point, and the observed associations may therefore be affected by shared measurement method. Shared method variance could inflate the associations among self-reported resources and outcomes, so the reported coefficients should not be read as precise estimates of the strength of these relationships. Future studies could reduce this risk through temporal separation of predictor and outcome measurement, multi-source data such as institutional records of AI training or access, and a reliable marker or latent method factor.

Third, the scale requires further psychometric strengthening. Although the overall AITA alpha was 0.858, alpha does not establish unidimensionality. All reported core constructs had AVE below 0.50, ITI alpha was 0.611, and the marker-variable alpha was 0.438; the EFA also did not clearly recover four dimensions and HTMT indicated substantial overlap. The current evidence therefore supports only provisional use of the overall score. The present AITA-only comparison did not show a meaningful fit advantage for the correlated four-factor model. Model fit alone does not establish construct validity, so future studies should compare one-factor, correlated four-factor, second-order, and bifactor models with larger samples and test measurement invariance across samples. Fourth, the available adaptation record does not include a dated translation/back-translation protocol or a separate pilot-testing record, so semantic equivalence remains uncertain. Geographic region was not collected, and the survey was distributed through forwarding channels without a recorded invitation denominator; regional coverage and a formal response rate therefore cannot be assessed. Limited prior exposure was present in both samples: in Sample A, 27 of 300 participants reported never using AI, and in Sample B, 34 of 495 (6.9%) reported never using AI and 162 (32.7%) reported no prior international/intercultural experience. Some context-specific responses may therefore reflect anticipated or perceived readiness rather than direct experience, and the associations involving these measures should be interpreted with this mix of experience levels in mind; this interpretation is tentative and requires direct validation, for example by comparing results across levels of intercultural teaching involvement. The two samples were collected in separate time windows, but because the survey was anonymous, the absence of individual-level overlap could not be verified. In addition, job demands such as workload were not measured, which limits the explanation of emotional exhaustion within the JD-R framework.

Fifth, the moderating role of institutional support was not supported. This may indicate that institutional support is closer to a direct organizational resource than to a boundary condition between AI acceptance and self-efficacy. Future research could distinguish among different forms of support, such as technical support, ethical support, emotional support, resource support, and policy support, to examine which types of support are associated with stronger links between AI acceptance and teacher resources.

Sixth, this study focused on university teachers in China. Whether the findings apply to other countries, regions, or educational systems remains to be tested. Intercultural teaching is shaped by institutional environments, levels of internationalization, AI policies, and teacher development systems. Cross-regional or cross-national comparative research would be valuable for further examining the applicability of the scale and the structural association model.

## 6 Conclusion

This study provides preliminary evidence for a context-specific overall measure of AI acceptance in intercultural teaching and reports cross-sectional associations with teacher resources and occupational-health indicators. AI acceptance was associated with self-efficacy and autonomous motivation, while self-efficacy showed the clearest association with both occupational wellbeing and emotional exhaustion. These findings are not causal and do not establish mediation or intervention effectiveness. Further psychometric and longitudinal research is needed.

The findings suggest that AI acceptance is associated with teacher resources that are, in turn, associated with occupational-health indicators; this pattern does not establish mediation. Because the data were cross-sectional and the AITA subdimensions were not empirically distinct, the overall AITA score should be regarded as a preliminary measure requiring further validation.

## Data Availability

The raw data supporting the conclusions of this article will be made available by the authors, without undue reservation.
