# Supplementary material for: Development and preliminary validation of an AI acceptance scale for intercultural teaching and its cross-sectional associations with university teachers’ occupational wellbeing
Source: Front Public Health. 2026 Oct 28;14:1986187. doi: 10.3389/fpubh.2026.1986187 (PMC13752047; doi:10.3389/fpubh.2026.1986187)
Supplement: Supplementary file 1 [file Data_Sheet_1.DOCX]

**Supplementary Material**

**Development and Preliminary Validation of an AI Acceptance Scale for Intercultural Teaching and Its Cross-Sectional Associations With University Teachers' Occupational Well-Being**

Qi Zhang, Mohammad Ismath Ramzy Mohammad Ismail*, Abd Razak Bin Zakaria

** Correspondence: Mohammad Ismath Ramzy Mohammad Ismail, nhxe2391@outlook.com*

This file contains Supplementary Figure S1 and Supplementary Tables S1–S6 cited in the manuscript. Abbreviations: AITA = AI acceptance for intercultural teaching; PU = perceived usefulness; PEOU = perceived ease of use; ATT = attitude; BI = behavioral intention; ITI = intercultural teaching involvement; ITSE = intercultural teaching self-efficacy; ATM = autonomous teaching motivation; OWB = occupational well-being; EE = emotional exhaustion; PIS = perceived institutional support; MV = marker variable.

# 1 Supplementary Figure

**Supplementary Figure S1.** Exploratory simple slopes for perceived institutional support. Lines show the standardized simple slopes implied by the mean-centered moderation model reported in Table 8: beta = 0.192 at low PIS (-1 SD), beta = 0.216 at mean PIS, and beta = 0.240 at high PIS (+1 SD), based on the main-effect coefficient beta = 0.216 and interaction coefficient beta = 0.024. The vertical axis shows the AITA-related component of predicted intercultural teaching self-efficacy (simple slope × standardized AITA); the PIS main-effect offset (beta = 0.179) is omitted so that the three lines differ only in slope. Both axes are expressed in standard-deviation units (standardized scores). The AITA x PIS interaction was not significant (p = .588); the plot is therefore descriptive rather than evidence of a supported moderation effect.

# 2 Supplementary Tables

**Supplementary Table S1.** Item-level missingness in Sample B (15 items with the highest missing percentages).

| **Item** | **Missing n** | **Missing (%)** |
| --- | --- | --- |
| I8 | 46 | 9.293 |
| H4 | 30 | 6.061 |
| I5 | 28 | 5.657 |
| I3 | 25 | 5.051 |
| H5 | 24 | 4.848 |
| I7 | 21 | 4.242 |
| J3 | 19 | 3.838 |
| J4 | 19 | 3.838 |
| F6 | 17 | 3.434 |
| G6 | 15 | 3.030 |
| I4 | 13 | 2.626 |
| J1 | 12 | 2.424 |
| E6 | 12 | 2.424 |
| E3 | 11 | 2.222 |
| H3 | 11 | 2.222 |

*Note.* Cleaned Sample B, N = 495. Only the 15 items with the highest proportions of missing responses are shown. Missing responses were dispersed across cases: 253 of the 495 cases (51.1%) had at least one missing response among the 51 measurement items, and 242 cases were complete.

**Supplementary Table S2.** Multiple-imputation-pooled CFA and SEM fit summaries in Sample B.

| **Model** | **n** | **χ²** | **df** | **χ²/df** | **CFI** | **TLI** | **RMSEA** |
| --- | --- | --- | --- | --- | --- | --- | --- |
| CFA-11F | 495 | 1305.953 | 1169 | 1.117 | 0.973 | 0.971 | 0.015 |
| SEM-1AITA | 495 | 1218.517 | 1018 | 1.197 | 0.960 | 0.958 | 0.020 |

*Note.* Estimates are pooled across five imputed data sets (robust maximum likelihood). CFA-11F denotes the 11-factor CFA using 51 observed items (C1-C4, D1-D12, E1-E6, F1-F6, G1-G6, H1-H5, I1-I8, and J1-J4), including the four marker items. SEM-1AITA denotes the latent SEM using the 47 substantive items C1-I8, excluding J1-J4, with one overall AITA factor plus ITSE, ATM, OWB, EE, PIS, and ITI and the prespecified structural paths. The CFA and SEM rows are not a nested-model comparison. With 47 observed items, there are 47 x 48 / 2 = 1,128 observed moments; the SEM has 110 free parameters, yielding df = 1,018. Therefore, the reported df values (1169 and 1018) and chi-square values should not be interpreted as if one model were obtained by simply adding paths to the other. Across the 51 item columns used in measurement analyses, 242 cases were complete before imputation; the MI analyses retained all 495 cases. The SEM fixed the residual covariances ITSE–ATM and OWB–EE to zero; because the OWB–EE constraint is restrictive, the SEM is reported only as a global-fit check, and its path estimates were not used for hypothesis testing.

**Supplementary Table S3.** Survey item wording used in the questionnaire. ATM items F1–F6 were presented after the stem “I engage in intercultural/internationalized teaching because…”.

| **Construct** | **Item** | **English wording** |
| --- | --- | --- |
| ITI | C1 | In the past academic year, I taught international students, Sino-foreign cooperative programs, or internationalized courses. |
| ITI | C2 | I conducted virtual exchange or online international collaborative learning, such as COIL. |
| ITI | C3 | My courses included intercultural or internationalized content, such as bilingual teaching, intercultural cases, or country comparisons. |
| ITI | C4 | I collaborated with overseas teachers, students, or institutions in teaching. |
| AITA-PU | D1 | Using AI tools for intercultural teaching improves my teaching efficiency. |
| AITA-PU | D2 | These AI tools help me accomplish intercultural teaching tasks better. |
| AITA-PU | D3 | Overall, these AI tools are useful for my intercultural teaching. |
| AITA-PEOU | D4 | I can easily learn to use these AI tools for intercultural teaching. |
| AITA-PEOU | D5 | Operating these AI tools is clear and understandable to me. |
| AITA-PEOU | D6 | Becoming skillful with these AI tools does not require too much extra effort for me. |
| AITA-ATT | D7 | I think using AI tools in intercultural teaching is a good choice. |
| AITA-ATT | D8 | I hold a positive attitude toward using AI tools in intercultural teaching. |
| AITA-ATT | D9 | Reasonable use of AI tools in intercultural teaching can improve my teaching experience. |
| AITA-BI | D10 | I intend to continue using AI tools in my future intercultural teaching. |
| AITA-BI | D11 | When conditions allow, I will use these AI tools more frequently in my teaching. |
| AITA-BI | D12 | I would recommend the reasonable use of AI tools in intercultural teaching to my colleagues. |
| ITSE | E1 | I am confident that I can teach effectively in international or cross-border settings. |
| ITSE | E2 | I can adapt my teaching to students from different cultural backgrounds. |
| ITSE | E3 | I can handle cultural differences and misunderstandings in intercultural teaching. |
| ITSE | E4 | I am able to design teaching activities that fit intercultural learning needs. |
| ITSE | E5 | I can communicate effectively when collaborating with international students or overseas partners. |
| ITSE | E6 | Overall, I am confident in my intercultural teaching competence. |
| ATM | F1 | I find intercultural teaching enjoyable in itself. |
| ATM | F2 | Intercultural teaching is interesting and meaningful to me. |
| ATM | F3 | I like exploring how to teach intercultural courses better. |
| ATM | F4 | Intercultural teaching is personally important to me. |
| ATM | F5 | Teaching intercultural courses well aligns with my professional values. |
| ATM | F6 | I identify with the meaning and value of intercultural/internationalized teaching. |
| OWB | G1 | I gain a sense of accomplishment from my work as a teacher. |
| OWB | G2 | I feel identification with and belonging to my teaching profession. |
| OWB | G3 | Overall, I am satisfied with my professional life. |
| OWB | G4 | My work makes me feel fulfilled and meaningful. |
| OWB | G5 | I often experience positive emotions at work. |
| OWB | G6 | My overall well-being is good, and I can cope with work demands relatively calmly. |
| EE | H1 | My teaching work often makes me feel emotionally depleted. |
| EE | H2 | At the end of a workday, I often feel physically and mentally exhausted. |
| EE | H3 | I feel tired when I think about starting another day of teaching and related work. |
| EE | H4 | Continuous teaching, communication, and management tasks make me feel highly stressed. |
| EE | H5 | I sometimes feel that my work energy is nearly used up. |
| PIS | I1 | My institution values and supports teachers' intercultural/internationalized teaching. |
| PIS | I2 | My institution encourages teachers to use AI tools appropriately to improve intercultural/internationalized teaching. |
| PIS | I3 | My institution provides necessary training or guidance for teachers to use AI in intercultural teaching. |
| PIS | I4 | My institution provides necessary resources and conditions for intercultural teaching, such as platforms, funding, course support, or international collaboration channels. |
| PIS | I5 | When I face technical, ethical, or administrative difficulties in AI-enabled intercultural teaching, my institution is willing to help. |
| PIS | I6 | My institution cares about teachers' professional development in integrating AI with internationalized teaching. |
| PIS | I7 | My institution recognizes teachers' efforts and achievements in intercultural teaching and AI-based teaching innovation. |
| PIS | I8 | Overall, I can genuinely feel my institution's support for AI-supported intercultural teaching. |

**Supplementary Table S4.** Scale sources, adaptation domains, and scoring record.

| **Construct** | **Items** | **Source/adaptation record** | **Domain distinction and scoring** |
| --- | --- | --- | --- |
| ITI | C1-C4 | Self-developed frequency/breadth indicator | Intercultural/internationalized teaching involvement; frequency score 1-5; contextual covariate. |
| AITA | D1-D12 | Self-developed from TAM | PU, PEOU, ATT, and BI in intercultural teaching; 5-point agreement score. |
| ITSE | E1-E6 | Contextualized adaptation based on Romijn et al. (2021) and Deardorff (2006) | Capability beliefs about internationalized/cross-border teaching; 5-point agreement score. |
| ATM | F1-F6 | Adapted from WTMST (Fernet et al., 2008), informed by SDT (Deci & Ryan, 2000) | Intrinsic and identified regulation for teaching; 5-point agreement score; distinct from capability and well-being. |
| OWB | G1-G6 | Adapted from PERMA-Teacher (Kern et al., 2014) and Warr (1990) | Occupational meaning, achievement, positive affect, and satisfaction; 5-point agreement score. |
| EE | H1-H5 | Contextualized emotional-exhaustion short form based on MBI-ES (Maslach & Jackson, 1981; Maslach et al., 1996) | Exhaustion symptoms; frequency score 1-5; separate strain outcome. |
| PIS | I1-I8 | Contextualized POS/SPOS adaptation; Eisenberger et al. (1986) | Institutional resources, training, technical support, policy recognition, and development support; 5-point agreement score. |
| MV | J1-J4 | Self-developed neutral work-habit marker | Used only for common-method-bias diagnostics; 5-point agreement score. |

*Note.* This table reports the source/adaptation record and contextual domains. It does not reproduce copyrighted source-item wording.

*Scoring note.* Unless otherwise specified, construct scores were arithmetic means of available items. The core attitude/resource constructs used 1 = strongly disagree to 5 = strongly agree. ITI used 1 = never to 5 = very frequently, and EE used 1 = never to 5 = always. The current questionnaire version contained no reverse-keyed core items.

*Source-record note.* The citation is standardized as Romijn et al. (2021). PIS is consistently treated as an eight-item construct (I1-I8). The measures are reported as contextualized adaptations, and the original copyrighted item wording is not reproduced.

**Supplementary Table S5.** AITA-only CFA model comparison in Sample B.

| **Model** | **Scaled χ²** | **df** | **CFI** | **TLI** | **RMSEA** | **SRMR** |
| --- | --- | --- | --- | --- | --- | --- |
| One-factor AITA | 49.70 | 54 | 1.000 | 1.004 | 0.000 | 0.023 |
| Correlated four-factor AITA | 45.49 | 48 | 1.000 | 1.003 | 0.000 | 0.022 |

*Note.* Both models were estimated with robust maximum likelihood and full-information maximum likelihood for item-level missingness in cleaned Sample B (n = 495). The comparison is a sensitivity analysis; similarly close fit does not establish that the four AITA facets are empirically distinct.

**Supplementary Table S6.** Comparison of structural path estimates between multiple-imputation and non-imputed score regressions in Sample B.

*Panel A.* Structural paths (standardized coefficients).

| **Outcome** | **Predictor** | **MI pooled β (SE)** | **p** | **Non-imputed β (SE)** | **p** |
| --- | --- | --- | --- | --- | --- |
| ITSE | AITA | 0.201 (0.044) | < .001 | 0.200 (0.044) | < .001 |
| ITSE | PIS | 0.154 (0.045) | < .001 | 0.154 (0.045) | < .001 |
| ITSE | ITI | 0.155 (0.043) | < .001 | 0.153 (0.043) | < .001 |
| ITSE | AITA × PIS | 0.026 (0.046) | .565 | 0.027 (0.045) | .548 |
| ATM | AITA | 0.178 (0.045) | < .001 | 0.180 (0.044) | < .001 |
| ATM | ITI | 0.100 (0.045) | .026 | 0.096 (0.044) | .031 |
| OWB | AITA | 0.022 (0.044) | .617 | 0.022 (0.044) | .618 |
| OWB | ITSE | 0.263 (0.045) | < .001 | 0.263 (0.045) | < .001 |
| OWB | ATM | 0.197 (0.044) | < .001 | 0.199 (0.044) | < .001 |
| OWB | ITI | −0.009 (0.043) | .838 | −0.009 (0.043) | .841 |
| EE | AITA | −0.051 (0.046) | .273 | −0.053 (0.046) | .249 |
| EE | ITSE | −0.155 (0.048) | .001 | −0.154 (0.047) | .001 |
| EE | ATM | −0.084 (0.046) | .069 | −0.080 (0.046) | .083 |
| EE | ITI | −0.048 (0.046) | .302 | −0.045 (0.045) | .325 |

*Panel B.* Indirect associations.

| **Indirect association** | **MI estimate*** | **Non-imputed estimate** | **Non-imputed 95% bootstrap CI** |
| --- | --- | --- | --- |
| AITA → ITSE → OWB | 0.053 | 0.053 | [0.028, 0.083] |
| AITA → ATM → OWB | 0.035 | 0.036 | [0.016, 0.061] |
| AITA → ITSE → EE | −0.031 | −0.031 | [−0.058, −0.009] |
| AITA → ATM → EE | −0.015 | −0.014 | [−0.035, 0.001] |
| Moderated indirect: ITSE → OWB | 0.007 | 0.007 | [−0.013, 0.029] |
| Moderated indirect: ITSE → EE | −0.004 | −0.004 | [−0.019, 0.008] |

*Note.* MI = multiple imputation. MI direct-path estimates (Panel A) are standardized scale-score regression coefficients pooled across five imputed datasets using Rubin's rules (p values based on Barnard–Rubin degrees of freedom). Non-imputed estimates are the primary score-based regressions reported in Table 7 (N = 495; scale scores were means of available items and were non-missing for all cases). The value n = 242 refers only to the item-level complete-case 51-item measurement analysis and is not the sample size for this table. Non-imputed bootstrap confidence intervals are the percentile intervals reported in Table 7 (5,000 resamples; seed 20260701). *MI indirect-association estimates were calculated within each imputed dataset and then summarized as the arithmetic mean of the five imputation-specific products; no formal Rubin- or D2-pooled confidence interval was calculated for these products, and they were not combined with the non-imputed bootstrap intervals.
