# Supplementary material for: Development and preliminary validation of an AI acceptance scale for intercultural teaching and its cross-sectional associations with university teachers’ occupational wellbeing
Source: Front Public Health. 2026 Oct 28;14:1986187. doi: 10.3389/fpubh.2026.1986187 (PMC13752047; doi:10.3389/fpubh.2026.1986187)
Supplement: Supplementary file 2 [file Data_Sheet_2.DOCX]

**Analysis Code Appendix**

*Development and Preliminary Validation of an AI Acceptance Scale for Intercultural Teaching and Its Cross-Sectional Associations With University Teachers' Occupational Well-Being*

# Scope

This appendix reproduces the analysis syntax and model specifications used in the revision. It contains no participant-level data.

- **Listing 1 (SPSS native analysis syntax.sps).** Documents the SPSS-native EFA, reliability, descriptive, correlation, and score-based regression steps.
- **Listing 2 (Latent model specification.R).** Is the single source of the item-level CFA and latent-SEM specifications. Listing 3 loads it with source(), so it must be saved under this file name in the working directory before Listing 3 is run.
- **Listing 3 (Run latent sensitivity.R).** run_latent_sensitivity.R is the runnable R script. After the analyst sets a local de-identified data path, it performs five-imputation MLR CFA/SEM with seed 20260701 and explicitly requests D2 pooling for the imputation-based model-test summaries. It also computes the 5,000-resample score-based indirect-association bootstrap summaries. The bootstrap standardizes each resample, reports the full-sample point estimate, and includes the two moderated-indirect indices shown in Table 7. The bootstrap summaries are intentionally kept separate from the MI/D2 model tests and are not presented as formal D2-pooled bootstrap intervals.

The script also contains the FIML one-factor versus correlated four-factor AITA comparison for Supplementary Table S5, the complete-case CR/AVE/loading diagnostics for Table 5 (CR and AVE calculated from standardized loadings with the Fornell and Larcker formulas), and an explicit pairwise HTMT diagnostic for Table 6 (the arithmetic-mean HTMT of Henseler et al., 2015, computed from absolute item correlations so that values are positive for negatively related constructs such as EE). These diagnostics are coded directly rather than called from semTools, whose default functions use different variance and HTMT2 definitions. The SPSS syntax contains the Harman single-factor extraction used for the common-method-bias diagnostic. These sections document the calculation rules; the submission does not claim that one script regenerates every table-formatting operation or every imputation-specific sensitivity output.

The SEM syntax explicitly fixes the residual covariances ITSE ~~ ATM and OWB ~~ EE to zero. This reproduces the reported 110 free parameters and df = 1,018 rather than allowing additional residual covariances in the fitted specification. Because the OWB–EE constraint is restrictive, the manuscript uses the latent SEM only as a global-fit check; its path estimates are not used for hypothesis testing.

The manuscript reports the software versions used for the sensitivity analyses (R 4.6.1, lavaan 0.6.21, lavaan.mi 0.1.0, and mice 3.19.0). Response-level data are not included in the submission package because the survey concerns teachers' occupational experiences and institutional contexts. The de-identified data and complete analysis records can be requested from the corresponding author subject to institutional and ethical restrictions.

# Listing 1. SPSS native analysis syntax.sps

```
* Submission-safe SPSS syntax for the native analyses.
* Replace the two relative file names (Sample A cleaned.sav, Sample B cleaned.sav)
* with local paths after opening SPSS.
* No participant-level data are distributed with this syntax.

SET UNICODE=ON.
SET DECIMAL=DOT.

TITLE 'Sample A: EFA for AI acceptance items'.
GET FILE='Sample A cleaned.sav'.
DATASET NAME SampleA.
FACTOR
  /VARIABLES D1 D2 D3 D4 D5 D6 D7 D8 D9 D10 D11 D12
  /MISSING LISTWISE
  /ANALYSIS D1 D2 D3 D4 D5 D6 D7 D8 D9 D10 D11 D12
  /PRINT INITIAL EXTRACTION KMO ROTATION
  /FORMAT SORT BLANK(.30)
  /CRITERIA FACTORS(4) ITERATE(25)
  /EXTRACTION ML
  /ROTATION OBLIMIN
  /METHOD=CORRELATION.

TITLE 'Sample B: Reliability, scale scoring, and descriptive statistics'.
GET FILE='Sample B cleaned.sav'.
DATASET NAME SampleB.

* Scale scores are arithmetic means of available items, as reported in Methods.
COMPUTE ITI=MEAN(C1 TO C4).
COMPUTE AITA=MEAN(D1 TO D12).
COMPUTE PU=MEAN(D1 TO D3).
COMPUTE PEOU=MEAN(D4 TO D6).
COMPUTE ATT=MEAN(D7 TO D9).
COMPUTE BI=MEAN(D10 TO D12).
COMPUTE ITSE=MEAN(E1 TO E6).
COMPUTE ATM=MEAN(F1 TO F6).
COMPUTE OWB=MEAN(G1 TO G6).
COMPUTE EE=MEAN(H1 TO H5).
COMPUTE PIS=MEAN(I1 TO I8).
COMPUTE MV=MEAN(J1 TO J4).
EXECUTE.

RELIABILITY
  /VARIABLES=C1 C2 C3 C4
  /SCALE('ITI') ALL /MODEL=ALPHA
  /STATISTICS=DESCRIPTIVE SCALE CORR.
RELIABILITY
  /VARIABLES=D1 D2 D3
  /SCALE('PU') ALL /MODEL=ALPHA
  /STATISTICS=DESCRIPTIVE SCALE CORR.
RELIABILITY
  /VARIABLES=D4 D5 D6
  /SCALE('PEOU') ALL /MODEL=ALPHA
  /STATISTICS=DESCRIPTIVE SCALE CORR.
RELIABILITY
  /VARIABLES=D7 D8 D9
  /SCALE('ATT') ALL /MODEL=ALPHA
  /STATISTICS=DESCRIPTIVE SCALE CORR.
RELIABILITY
  /VARIABLES=D10 D11 D12
  /SCALE('BI') ALL /MODEL=ALPHA
  /STATISTICS=DESCRIPTIVE SCALE CORR.
RELIABILITY
  /VARIABLES=D1 D2 D3 D4 D5 D6 D7 D8 D9 D10 D11 D12
  /SCALE('AITA') ALL /MODEL=ALPHA
  /STATISTICS=DESCRIPTIVE SCALE CORR.
RELIABILITY
  /VARIABLES=E1 E2 E3 E4 E5 E6
  /SCALE('ITSE') ALL /MODEL=ALPHA
  /STATISTICS=DESCRIPTIVE SCALE CORR.
RELIABILITY
  /VARIABLES=F1 F2 F3 F4 F5 F6
  /SCALE('ATM') ALL /MODEL=ALPHA
  /STATISTICS=DESCRIPTIVE SCALE CORR.
RELIABILITY
  /VARIABLES=G1 G2 G3 G4 G5 G6
  /SCALE('OWB') ALL /MODEL=ALPHA
  /STATISTICS=DESCRIPTIVE SCALE CORR.
RELIABILITY
  /VARIABLES=H1 H2 H3 H4 H5
  /SCALE('EE') ALL /MODEL=ALPHA
  /STATISTICS=DESCRIPTIVE SCALE CORR.
RELIABILITY
  /VARIABLES=I1 I2 I3 I4 I5 I6 I7 I8
  /SCALE('PIS') ALL /MODEL=ALPHA
  /STATISTICS=DESCRIPTIVE SCALE CORR.
RELIABILITY
  /VARIABLES=J1 J2 J3 J4
  /SCALE('MV') ALL /MODEL=ALPHA
  /STATISTICS=DESCRIPTIVE SCALE CORR.

DESCRIPTIVES VARIABLES=AITA PU PEOU ATT BI ITSE ATM OWB EE PIS ITI MV
  /STATISTICS=MEAN STDDEV MIN MAX SKEWNESS KURTOSIS.

TITLE 'Harman single-factor diagnostic (Section 4.1 common-method-bias check)'.
FACTOR
  /VARIABLES C1 C2 C3 C4 D1 D2 D3 D4 D5 D6 D7 D8 D9 D10 D11 D12
             E1 E2 E3 E4 E5 E6 F1 F2 F3 F4 F5 F6 G1 G2 G3 G4 G5 G6
             H1 H2 H3 H4 H5 I1 I2 I3 I4 I5 I6 I7 I8 J1 J2 J3 J4
  /MISSING LISTWISE
  /ANALYSIS C1 C2 C3 C4 D1 D2 D3 D4 D5 D6 D7 D8 D9 D10 D11 D12
             E1 E2 E3 E4 E5 E6 F1 F2 F3 F4 F5 F6 G1 G2 G3 G4 G5 G6
             H1 H2 H3 H4 H5 I1 I2 I3 I4 I5 I6 I7 I8 J1 J2 J3 J4
  /PRINT INITIAL EXTRACTION
  /CRITERIA FACTORS(1) ITERATE(25)
  /EXTRACTION ML
  /ROTATION NOROTATE
  /METHOD=CORRELATION.

TITLE 'Sample B: Pearson correlations for scale scores'.
CORRELATIONS
  /VARIABLES=AITA ITSE ATM OWB EE PIS ITI MV PU PEOU ATT BI
  /PRINT=TWOTAIL NOSIG
  /MISSING=PAIRWISE.

* Mean-centering and the interaction term are computed from the scale scores.
* The same centered interaction is used in both specifications. Table 7
* adjusts for ITI; Table 8 is the centered moderation summary without ITI.
AGGREGATE
  /OUTFILE=* MODE=ADDVARIABLES
  /BREAK=
  /mean_AITA=MEAN(AITA)
  /mean_PIS=MEAN(PIS).
COMPUTE c_AITA=AITA-mean_AITA.
COMPUTE c_PIS=PIS-mean_PIS.
COMPUTE AITA_x_PIS=c_AITA*c_PIS.
EXECUTE.

TITLE 'Table 7: Primary score-based structural associations'.
REGRESSION
  /MISSING LISTWISE
  /STATISTICS COEFF OUTS R ANOVA CI(95) COLLIN
  /DEPENDENT ITSE
  /METHOD=ENTER AITA PIS ITI AITA_x_PIS.
REGRESSION
  /MISSING LISTWISE
  /STATISTICS COEFF OUTS R ANOVA CI(95) COLLIN
  /DEPENDENT ATM
  /METHOD=ENTER AITA ITI.
REGRESSION
  /MISSING LISTWISE
  /STATISTICS COEFF OUTS R ANOVA CI(95) COLLIN
  /DEPENDENT OWB
  /METHOD=ENTER AITA ITSE ATM ITI.
REGRESSION
  /MISSING LISTWISE
  /STATISTICS COEFF OUTS R ANOVA CI(95) COLLIN
  /DEPENDENT EE
  /METHOD=ENTER AITA ITSE ATM ITI.

TITLE 'Table 8: Centered moderation summary without ITI covariate'.
REGRESSION
  /MISSING LISTWISE
  /STATISTICS COEFF OUTS R ANOVA CI(95) COLLIN
  /DEPENDENT ITSE
  /METHOD=ENTER c_AITA c_PIS AITA_x_PIS.

* Save a local working copy only if an analyst needs to inspect computed fields.
* SAVE OUTFILE='Sample B scored local.sav'.
```

# Listing 2. Latent model specification.R

```
# Latent sensitivity-model specifications used in the revision.
# This file contains no participant-level data and does not perform estimation.

# AITA-only one-factor and correlated four-factor models (Supplementary Table S5)
aita_one_factor <- '
AITA =~ D1 + D2 + D3 + D4 + D5 + D6 + D7 + D8 + D9 + D10 + D11 + D12
'

aita_correlated_four_factor <- '
PU   =~ D1 + D2 + D3
PEOU =~ D4 + D5 + D6
ATT  =~ D7 + D8 + D9
BI   =~ D10 + D11 + D12
'

# Pooled 11-factor CFA: 51 items, including marker items J1-J4.
cfa_11_factor <- '
ITI  =~ C1 + C2 + C3 + C4
PU   =~ D1 + D2 + D3
PEOU =~ D4 + D5 + D6
ATT  =~ D7 + D8 + D9
BI   =~ D10 + D11 + D12
ITSE =~ E1 + E2 + E3 + E4 + E5 + E6
ATM  =~ F1 + F2 + F3 + F4 + F5 + F6
OWB  =~ G1 + G2 + G3 + G4 + G5 + G6
EE   =~ H1 + H2 + H3 + H4 + H5
PIS  =~ I1 + I2 + I3 + I4 + I5 + I6 + I7 + I8
MV   =~ J1 + J2 + J3 + J4
'

# Latent SEM: 47 substantive items, excluding marker items J1-J4.
# The structural paths are prespecified associations, not causal effects.
sem_1_aita <- '
AITA =~ D1 + D2 + D3 + D4 + D5 + D6 + D7 + D8 + D9 + D10 + D11 + D12
ITI  =~ C1 + C2 + C3 + C4
ITSE =~ E1 + E2 + E3 + E4 + E5 + E6
ATM  =~ F1 + F2 + F3 + F4 + F5 + F6
OWB  =~ G1 + G2 + G3 + G4 + G5 + G6
EE   =~ H1 + H2 + H3 + H4 + H5
PIS  =~ I1 + I2 + I3 + I4 + I5 + I6 + I7 + I8
ITSE ~ AITA + PIS + ITI
ATM  ~ AITA + ITI
OWB  ~ AITA + ITSE + ATM + ITI
EE   ~ AITA + ITSE + ATM + ITI
# Residual covariances between endogenous latent outcomes are not freely
# estimated in the reported SEM parameterization.
ITSE ~~ 0*ATM
OWB ~~ 0*EE
'
```

# Listing 3. Run latent sensitivity.R

```
# Reproducible sensitivity and indirect-association analysis.
# No participant-level data are included. Run this script from a working
# directory that contains this file, Latent model specification.R, and a local,
# de-identified Sample B cleaned.csv file (not distributed).

required <- c("lavaan", "lavaan.mi", "mice")
missing <- required[!vapply(required, requireNamespace, logical(1), quietly = TRUE)]
if (length(missing)) stop("Install required packages: ", paste(missing, collapse = ", "))
suppressPackageStartupMessages({ library(lavaan); library(lavaan.mi); library(mice) })

spec_path <- "Latent model specification.R"
if (!file.exists(spec_path)) stop("Place Latent model specification.R in the working directory or set spec_path.")
source(spec_path)

data_path <- "Sample B cleaned.csv"  # local analyst path; not distributed
if (!file.exists(data_path)) stop("Set data_path to the local de-identified Sample-B file.")
dat <- read.csv(data_path, check.names = FALSE)

item_cols <- c(paste0("C", 1:4), paste0("D", 1:12), paste0("E", 1:6),
               paste0("F", 1:6), paste0("G", 1:6), paste0("H", 1:5),
               paste0("I", 1:8), paste0("J", 1:4))
missing_items <- setdiff(item_cols, names(dat))
if (length(missing_items)) stop("Missing item columns: ", paste(missing_items, collapse = ", "))
item_dat <- dat[, item_cols, drop = FALSE]

# Supplementary Table S5: AITA-only FIML comparison. The scaled MLR
# statistics are the reported chi-square/fit-index version.
aita_dat <- dat[, paste0("D", 1:12), drop = FALSE]
fit_aita_one_fiml <- lavaan::cfa(
  aita_one_factor, data = aita_dat, estimator = "MLR", missing = "fiml"
)
fit_aita_four_fiml <- lavaan::cfa(
  aita_correlated_four_factor, data = aita_dat,
  estimator = "MLR", missing = "fiml"
)
print(lavaan::fitMeasures(fit_aita_one_fiml,
                  c("chisq.scaled", "df", "cfi.scaled", "tli.scaled",
                    "rmsea.scaled", "srmr")))
print(lavaan::fitMeasures(fit_aita_four_fiml,
                  c("chisq.scaled", "df", "cfi.scaled", "tli.scaled",
                    "rmsea.scaled", "srmr")))

# Table 5 diagnostics: complete-case 11-factor CFA, standardized loading
# ranges, and the Fornell-Larcker CR/AVE formulas used for the displayed table.
fit_cfa_complete <- lavaan::cfa(
  cfa_11_factor, data = item_dat, estimator = "MLR", missing = "listwise"
)
std <- lavaan::parameterEstimates(fit_cfa_complete, standardized = TRUE)
constructs <- c("ITI", "PU", "PEOU", "ATT", "BI", "ITSE", "ATM",
                 "OWB", "EE", "PIS", "MV")
cr_ave <- do.call(rbind, lapply(constructs, function(x) {
  lam <- std$std.all[std$op == "=~" & std$lhs == x]
  cr <- sum(lam)^2 / (sum(lam)^2 + sum(1 - lam^2))
  ave <- mean(lam^2)
  data.frame(construct = x, CR = cr, AVE = ave,
             min_loading = min(lam), max_loading = max(lam))
}))
print(cr_ave)

# Table 6 diagnostic: arithmetic-mean HTMT (Henseler et al., 2015) using absolute pairwise item correlations. This explicit
# implementation keeps the correlation/missing-data rule visible to readers.
htmt_pairwise <- function(data, scale_map) {
  nm <- names(scale_map)
  out <- diag(length(nm)); dimnames(out) <- list(nm, nm)
  for (a in nm) for (b in nm) if (a != b) {
    ra <- cor(data[, scale_map[[a]], drop = FALSE], use = "pairwise.complete.obs")
    rb <- cor(data[, scale_map[[b]], drop = FALSE], use = "pairwise.complete.obs")
    cross <- cor(data[, c(scale_map[[a]], scale_map[[b]]), drop = FALSE],
                 use = "pairwise.complete.obs")
    hetero <- mean(abs(cross[scale_map[[a]], scale_map[[b]]]), na.rm = TRUE)
    mono_a <- mean(abs(ra[upper.tri(ra)]), na.rm = TRUE)
    mono_b <- mean(abs(rb[upper.tri(rb)]), na.rm = TRUE)
    out[a, b] <- hetero / sqrt(mono_a * mono_b)
  }
  out
}
htmt_scales <- list(
  PU = paste0("D", 1:3), PEOU = paste0("D", 4:6),
  ATT = paste0("D", 7:9), BI = paste0("D", 10:12),
  ITSE = paste0("E", 1:6), ATM = paste0("F", 1:6),
  OWB = paste0("G", 1:6), EE = paste0("H", 1:5),
  PIS = paste0("I", 1:8)
)
print(htmt_pairwise(dat, htmt_scales))

# Five imputations; parameter estimates use Rubin's rules, and model-test
# summaries are explicitly requested with D2 pooling below.
mi <- mice::mice(item_dat, m = 5, seed = 20260701, printFlag = FALSE)
fit_cfa <- lavaan.mi::cfa.mi(cfa_11_factor, data = mi, estimator = "MLR")
fit_sem <- lavaan.mi::sem.mi(sem_1_aita, data = mi, estimator = "MLR")
print(summary(fit_cfa, fit.measures = TRUE, standardized = TRUE,
              pool.method = "D2"))
print(summary(fit_sem, fit.measures = TRUE, standardized = TRUE,
              pool.method = "D2"))

# Score-based bootstrap. Each resample is standardized before fitting, and
# the interaction is then formed from standardized AITA and PIS. This matches
# the standardized-coefficient products reported in Table 7.
mean_items <- function(d, items) {
  z <- rowMeans(d[, items, drop = FALSE], na.rm = TRUE)
  z[rowSums(!is.na(d[, items, drop = FALSE])) == 0] <- NA_real_
  z
}
score_data <- data.frame(
  AITA = mean_items(dat, paste0("D", 1:12)),
  ITSE = mean_items(dat, paste0("E", 1:6)),
  ATM  = mean_items(dat, paste0("F", 1:6)),
  OWB  = mean_items(dat, paste0("G", 1:6)),
  EE   = mean_items(dat, paste0("H", 1:5)),
  PIS  = mean_items(dat, paste0("I", 1:8)),
  ITI  = mean_items(dat, paste0("C", 1:4))
)
score_data <- score_data[complete.cases(score_data), , drop = FALSE]

standardized_with_interaction <- function(d) {
  z <- as.data.frame(scale(d))
  z$AITA_x_PIS <- as.numeric(scale(z$AITA * z$PIS))
  z
}

indirect_once <- function(d) {
  z <- standardized_with_interaction(d)
  m_itse <- lm(ITSE ~ AITA + PIS + ITI + AITA_x_PIS, data = z)
  m_atm  <- lm(ATM  ~ AITA + ITI, data = z)
  m_owb  <- lm(OWB  ~ AITA + ITSE + ATM + ITI, data = z)
  m_ee   <- lm(EE   ~ AITA + ITSE + ATM + ITI, data = z)
  c(
    AITA_ITSE_OWB = unname(coef(m_itse)["AITA"] * coef(m_owb)["ITSE"]),
    AITA_ATM_OWB  = unname(coef(m_atm)["AITA"]  * coef(m_owb)["ATM"]),
    AITA_ITSE_EE  = unname(coef(m_itse)["AITA"] * coef(m_ee)["ITSE"]),
    AITA_ATM_EE   = unname(coef(m_atm)["AITA"]  * coef(m_ee)["ATM"]),
    moderated_ITSE_OWB = unname(coef(m_itse)["AITA_x_PIS"] * coef(m_owb)["ITSE"]),
    moderated_ITSE_EE  = unname(coef(m_itse)["AITA_x_PIS"] * coef(m_ee)["ITSE"])
  )
}

# The estimate column is the direct full-sample standardized point estimate,
# not the mean of the bootstrap replicates.
point_estimate <- indirect_once(score_data)
set.seed(20260701)
B <- 5000L
boot_mat <- replicate(
  B,
  indirect_once(score_data[sample.int(nrow(score_data), replace = TRUE), , drop = FALSE])
)
boot_summary <- t(vapply(names(point_estimate), function(nm) {
  x <- boot_mat[nm, ]
  c(estimate = unname(point_estimate[nm]),
    boot_se = sd(x, na.rm = TRUE),
    ci_low = unname(quantile(x, .025, na.rm = TRUE)),
    ci_high = unname(quantile(x, .975, na.rm = TRUE)))
}, numeric(4)))
print(boot_summary)

# The bootstrap summaries are separate from the MI/D2 model tests and are not
# formal D2-pooled bootstrap confidence intervals.
```
