# Supplementary material for: Development and preliminary validation of an AI acceptance scale for intercultural teaching and its cross-sectional associations with university teachers’ occupational wellbeing
Source: Front Public Health. 2026 Oct 28;14:1986187. doi: 10.3389/fpubh.2026.1986187 (PMC13752047; doi:10.3389/fpubh.2026.1986187)
Supplement: Supplementary file 3 [file Data_Sheet_3.DOCX]

**面向跨文化教学的人工智能接受度量表开发及其与高校教师职业幸福感的关联——跨文化教学自我效能与教学动机的作用、院校支持感知的调节**

**Development and Validation of an AI-for-Intercultural-Teaching Acceptance Scale and Its Associations with University Teachers' Occupational Well-Being: The Roles of Intercultural Teaching Self-Efficacy, Teaching Motivation, and Perceived Institutional Support**

正式施测版 / Formal Survey Version · 中英对照

修订说明：本文件为实际施测的问卷，标题为研究设计阶段的工作标题。论文将 AITA 量表报告为初步开发与初步验证，四个 TAM 维度仅作为暂定的内容维度。 Revision note: This is the questionnaire as administered; its title is the working title used at the design stage. The manuscript reports the AITA as undergoing preliminary development and initial validation, and treats the four TAM facets as provisional content facets.

# **一、卷首语与电子知情同意 Introduction and E-Consent**

尊敬的老师：您好！本问卷旨在了解高校教师在跨文化/国际化教学情境中对人工智能（AI）工具的接受度，并考察其与跨文化教学自我效能、教学动机、职业幸福感、职业倦怠及院校支持感知之间的关联。研究结果仅用于学术研究和整体统计分析。

本问卷为匿名填答，不收集姓名、手机号、身份证号等可识别个人身份的信息；数据将去标识化存储。参与完全自愿，您可在任何时候退出，且不会产生任何不利影响。预计填写时间约 10-12 分钟。

Dear teacher: This anonymous survey examines university teachers' acceptance of AI tools in intercultural/internationalized teaching and its associations with intercultural teaching self-efficacy, teaching motivation, occupational well-being, burnout, and perceived institutional support. The data will be used only for academic research and aggregate statistical analyses.

This survey does not collect personally identifiable information. Participation is voluntary, and you may withdraw at any time without any negative consequence. Estimated completion time is 10-12 minutes.

本问卷为研究工具附录。伦理审批信息以论文 Ethics Statement 中的正式记录为准，本附录不重复填写审批编号。

○ 我已阅读并理解上述内容，自愿参与本研究。

# **二、第一部分：筛查与基本信息 Part 1. Screening and Demographics**

| **题号** | **题目** | **选项** |
| --- | --- | --- |
| B1 | 您是否为中国高校在职专任教师，且近一学年承担实际授课任务？ | ○ 是 Yes ○ 否 No（选“否”则结束问卷） |
| B2 | 性别 Gender | ○ 男 ○ 女 ○ 其他 ○ 不愿透露 |
| B3 | 年龄 Age | ○ ≤30 ○ 31-40 ○ 41-50 ○ ≥51 |
| B4 | 教龄 Years of teaching | ○ ≤5 ○ 6-10 ○ 11-20 ○ ≥21 |
| B5 | 学科门类 Discipline | ○ 人文 ○ 社科 ○ 理工 ○ 医学 ○ 艺术 ○ 其他 |
| B6 | 院校类型 Institution type | ○ 研究型 ○ 教学研究型 ○ 教学型 ○ 其他 |
| B7 | 所在院校是否设有中外合作办学、国际合作项目或来华留学生教育项目？ | ○ 有 ○ 无 ○ 不清楚 |
| B8 | 既往国际/跨文化经历（留学、访学、国际项目、海外合作教学等） | ○ 无（None） ○ 1次（Once） ○ 2–3次（2-3 times） ○ 4次及以上（4+ times） |
| B9 | 过去一年，您在教学中使用 AI 工具的频率如何？ | ○ 从未（Never） ○ 偶尔（Occasionally） ○ 每月（Monthly） ○ 每周（Weekly） ○ 几乎每天（Almost daily） |
| B10 | 您在 AI 教学应用方面的培训/经验水平如何？ | ○ 低（Low） ○ 中低（Mid-low） ○ 中（Medium） ○ 中高（Mid-high） ○ 高（High） |

# **三、第二部分：量表测量 Part 2. Measures**

除特别说明外，以下题项均采用 5 点李克特量表：1 = 完全不同意，2 = 比较不同意，3 = 中立，4 = 比较同意，5 = 完全同意。请根据您的真实感受作答，无对错之分。

Unless otherwise noted, all items use a 5-point Likert scale: 1 = strongly disagree, 2 = somewhat disagree, 3 = neutral, 4 = somewhat agree, 5 = strongly agree.

本文中的“AI 工具”特指可支持跨文化/国际化教学的人工智能工具，例如 AI 翻译与润色、跨文化案例生成、智能教学助手、国际虚拟协作/COIL 平台中的 AI 功能、面向国际学生的学习支持工具等。

## **（一）跨文化教学卷入度 Intercultural Teaching Involvement**

量表来源：自编量表，用于刻画教师参与跨文化/国际化教学的实际程度，作为连续协变量纳入分析。

计分方式：频率计分，1 = 从不，2 = 偶尔，3 = 有时，4 = 经常，5 = 非常频繁。

| **编号** | **题项（中文）** | **Item (English)** |
| --- | --- | --- |
| C1 | 近一学年，我承担过面向国际学生、中外合作项目或国际化课程的教学任务。 | In the past academic year, I taught international students, Sino-foreign cooperative programs, or internationalized courses. |
| C2 | 我在教学中开展过国际虚拟交流或在线国际协作学习（如 COIL）。 | I conducted virtual exchange or online international collaborative learning, such as COIL. |
| C3 | 我的课程包含跨文化或国际化内容，如双语教学、跨文化案例、国别比较等。 | My courses included intercultural or internationalized content, such as bilingual teaching, intercultural cases, or country comparisons. |
| C4 | 我与境外教师、学生或机构在教学层面开展过合作。 | I collaborated with overseas teachers, students, or institutions in teaching. |

## **（二）面向跨文化教学的 AI 接受度 AI-for-Intercultural-Teaching Acceptance**

量表来源：自编焦点量表。题项基于技术接受模型（TAM）的四维结构生成，即感知有用性、感知易用性、使用态度和行为意向，并嵌入跨文化/国际化教学情境。本正式施测版已依据专家评审意见进行优化；各项来源、改编说明和分析结果见论文 Methods、Results 及参考文献。

计分方式：5 点同意度。

## **感知有用性 Perceived Usefulness (PU)**

| **编号** | **题项（中文）** | **Item (English)** |
| --- | --- | --- |
| D1 | 使用支持跨文化教学的 AI 工具能提升我的教学效率。 | Using AI tools for intercultural teaching improves my teaching efficiency. |
| D2 | 这类 AI 工具有助于我更好地完成跨文化教学任务。 | These AI tools help me accomplish intercultural teaching tasks better. |
| D3 | 总体而言，这类 AI 工具对我的跨文化教学是有用的。 | Overall, these AI tools are useful for my intercultural teaching. |

## **感知易用性 Perceived Ease of Use (PEOU)**

| **编号** | **题项（中文）** | **Item (English)** |
| --- | --- | --- |
| D4 | 我能较容易地学会使用这类支持跨文化教学的 AI 工具。 | I can easily learn to use these AI tools for intercultural teaching. |
| D5 | 对我而言，操作这类 AI 工具是清晰且易于理解的。 | Operating these AI tools is clear and understandable to me. |
| D6 | 熟练掌握这类 AI 工具对我来说不需要过多额外努力。 | Becoming skillful with these AI tools does not require too much extra effort for me. |

## **使用态度 Attitude Toward Use (ATT)**

| **编号** | **题项（中文）** | **Item (English)** |
| --- | --- | --- |
| D7 | 我认为在跨文化教学中使用 AI 工具是一个好的选择。 | I think using AI tools in intercultural teaching is a good choice. |
| D8 | 我对在跨文化教学中使用 AI 工具持积极态度。 | I hold a positive attitude toward using AI tools in intercultural teaching. |
| D9 | 在跨文化教学中合理使用 AI 工具有助于改善我的教学体验。 | Reasonable use of AI tools in intercultural teaching can improve my teaching experience. |

## **行为意向 Behavioral Intention (BI)**

| **编号** | **题项（中文）** | **Item (English)** |
| --- | --- | --- |
| D10 | 我打算在今后的跨文化教学中继续使用 AI 工具。 | I intend to continue using AI tools in my future intercultural teaching. |
| D11 | 在条件允许的情况下，我会更频繁地在教学中使用这类 AI 工具。 | When conditions allow, I will use these AI tools more frequently in my teaching. |
| D12 | 我愿意向同行推荐在跨文化教学中合理使用 AI 工具。 | I would recommend the reasonable use of AI tools in intercultural teaching to my colleagues. |

## **（三）跨文化教学自我效能 Intercultural Teaching Self-Efficacy**

量表来源：基于教师跨文化自我效能和跨文化能力相关文献进行情境化改编，题项明确锁定国际化/跨境教学情境，区别于一般多元文化课堂或通用教学自我效能。本正式施测版已依据专家评审意见进行优化；改编依据和测量说明见论文 Methods。

计分方式：5 点同意度。

| **编号** | **题项（中文）** | **Item (English)** |
| --- | --- | --- |
| E1 | 我有信心在国际化/跨境教学情境中有效组织教学。 | I am confident that I can teach effectively in international or cross-border settings. |
| E2 | 面对来自不同文化背景的学生，我能够灵活调整自己的教学方式。 | I can adapt my teaching to students from different cultural backgrounds. |
| E3 | 我能妥善处理跨文化教学中出现的文化差异与误解。 | I can handle cultural differences and misunderstandings in intercultural teaching. |
| E4 | 我有能力设计契合跨文化学习需求的教学活动。 | I am able to design teaching activities that fit intercultural learning needs. |
| E5 | 在与国际学生或境外伙伴协作时，我能够进行有效沟通。 | I can communicate effectively when collaborating with international students or overseas partners. |
| E6 | 总体而言，我对自己的跨文化教学能力充满信心。 | Overall, I am confident in my intercultural teaching competence. |

## **（四）教学动机（自主动机）Autonomous Teaching Motivation**

量表来源：基于自我决定理论和教师工作任务动机量表的自主动机维度进行情境化改编，聚焦内在动机和认同调节。为与研究模型一致，题项均限定为跨文化/国际化教学。本正式施测版已依据专家评审意见进行优化。

计分方式：5 点同意度。题干：我之所以投入跨文化/国际化教学，是因为...

| **编号** | **题项（中文）** | **Item (English)** |
| --- | --- | --- |
| F1 | ...我能从跨文化教学本身中获得乐趣。 | ...I find intercultural teaching enjoyable in itself. |
| F2 | ...跨文化教学让我感到有趣且富有意义。 | ...intercultural teaching is interesting and meaningful to me. |
| F3 | ...我喜欢探索如何把跨文化课程上得更好。 | ...I like exploring how to teach intercultural courses better. |
| F4 | ...跨文化教学对我个人而言很重要。 | ...intercultural teaching is personally important to me. |
| F5 | ...做好跨文化教学符合我的职业价值观。 | ...teaching intercultural courses well aligns with my professional values. |
| F6 | ...我认同跨文化/国际化教学的意义与价值。 | ...I identify with the meaning and value of intercultural/internationalized teaching. |

【注意力检测题 AC1】本题请直接选择“比较同意（4）”。 / Attention check: please select “Somewhat agree (4)”.

## **（五）职业幸福感 Occupational Well-Being**

量表来源：基于教师职业幸福感、PERMA 教师幸福感框架和工作相关情感幸福感理论进行情境化改编。为避免与自我效能构念重叠，题项聚焦职业认同、意义感、满意度、成就感、积极情绪和工作应对状态。本正式施测版已依据专家评审意见进行优化。

计分方式：5 点同意度。

| **编号** | **题项（中文）** | **Item (English)** |
| --- | --- | --- |
| G1 | 我从教师工作中获得成就感。 | I gain a sense of accomplishment from my work as a teacher. |
| G2 | 我对自己的教师职业感到认同与归属。 | I feel identification with and belonging to my teaching profession. |
| G3 | 总体而言，我对自己的职业生活感到满意。 | Overall, I am satisfied with my professional life. |
| G4 | 我的工作让我感到充实而有意义。 | My work makes me feel fulfilled and meaningful. |
| G5 | 在工作中，我常常体验到积极的情绪。 | I often experience positive emotions at work. |
| G6 | 我的身心状态总体良好，能够较从容地应对工作要求。 | My overall well-being is good, and I can cope with work demands relatively calmly. |

## **（六）职业倦怠：情绪耗竭 Emotional Exhaustion**

量表来源：基于教师职业倦怠中的情绪耗竭构念进行情境化改编。本部分为基于情绪耗竭构念的情境化改编题项；来源与改编说明见论文 Methods 和参考文献。

计分方式：频率计分，1 = 从不，2 = 很少，3 = 有时，4 = 经常，5 = 总是。

| **编号** | **题项（中文）** | **Item (English)** |
| --- | --- | --- |
| H1 | 教师工作常让我感到情绪上被消耗。 | My teaching work often makes me feel emotionally depleted. |
| H2 | 一天工作结束时，我常感到身心俱疲。 | At the end of a workday, I often feel physically and mentally exhausted. |
| H3 | 想到又要开始一天的教学和相关工作时，我会感到疲惫。 | I feel tired when I think about starting another day of teaching and related work. |
| H4 | 持续的教学、沟通和管理任务让我感到压力很大。 | Continuous teaching, communication, and management tasks make me feel highly stressed. |
| H5 | 我有时觉得自己的工作精力快被耗尽了。 | I sometimes feel that my work energy is nearly used up. |

【注意力检测题 AC2】本题请直接选择“很少（2）”。 / Attention check: please select “Rarely (2)”.

## **（七）院校支持感知 Perceived Institutional Support for AI-Enabled Intercultural Teaching**

量表来源：基于感知组织支持理论进行情境化改编，测量院校在“AI 支持的跨文化/国际化教学”方面提供的资源、培训、技术支持、政策认可和发展支持。该版本比一般“国际化教学支持”更贴合 H5/H6 的探索性调节路径。本正式施测版已依据专家评审意见进行优化。

计分方式：5 点同意度。

| **编号** | **题项（中文）** | **Item (English)** |
| --- | --- | --- |
| I1 | 我所在院校重视并支持教师开展跨文化/国际化教学。 | My institution values and supports teachers' intercultural/internationalized teaching. |
| I2 | 院校鼓励教师合理使用 AI 工具改进跨文化/国际化教学。 | My institution encourages teachers to use AI tools appropriately to improve intercultural/internationalized teaching. |
| I3 | 院校为教师使用 AI 开展跨文化教学提供必要的培训或指导。 | My institution provides necessary training or guidance for teachers to use AI in intercultural teaching. |
| I4 | 院校为跨文化教学提供必要的资源与条件，如平台、经费、课程支持或国际合作渠道。 | My institution provides necessary resources and conditions for intercultural teaching, such as platforms, funding, course support, or international collaboration channels. |
| I5 | 当我在 AI 支持的跨文化教学中遇到技术、伦理或管理困难时，院校愿意提供帮助。 | When I face technical, ethical, or administrative difficulties in AI-enabled intercultural teaching, my institution is willing to help. |
| I6 | 院校关心教师在 AI 与国际化教学融合方面的专业发展。 | My institution cares about teachers' professional development in integrating AI with internationalized teaching. |
| I7 | 院校认可教师在跨文化教学和 AI 教学创新方面的投入与成绩。 | My institution recognizes teachers' efforts and achievements in intercultural teaching and AI-based teaching innovation. |
| I8 | 总体而言，我能切实感受到院校对 AI 支持的跨文化教学的支持。 | Overall, I can genuinely feel my institution's support for AI-supported intercultural teaching. |

## **（八）共同方法偏差标记变量 Marker Variable for Common Method Bias**

量表来源：自编中性工作习惯题项。该变量与研究核心构念在理论上无直接关系，并在本研究中作为共同方法偏差诊断的标记变量。

计分方式：5 点同意度。

| **编号** | **题项（中文）** | **Item (English)** |
| --- | --- | --- |
| J1 | 我通常会把电子文件按日期或主题分类保存。 | I usually organize electronic files by date or topic. |
| J2 | 我习惯为常用文件设置清晰的文件名。 | I tend to give clear names to frequently used files. |
| J3 | 我会定期整理电脑桌面或云盘文件夹。 | I regularly organize my desktop or cloud folders. |
| J4 | 我倾向于把会议材料、课程材料或通知文件归档保存。 | I tend to archive meeting materials, course materials, or notification documents. |

# **附录 A：问卷构成与来源汇总 Appendix A. Composition and Sources**

| **构念 Construct** | **题数** | **来源 Source** | **维度** | **信效度阈值** |
| --- | --- | --- | --- | --- |
| 跨文化教学卷入度 ITI | 4 | Self-developed; frequency/breadth indicator | 单维 | alpha ≥ .70 |
| 跨文化教学 AI 接受度 AITA | 12 | Self-developed from TAM; PU/PEOU/ATT/BI in intercultural teaching | PU/PEOU/ATT/BI | alpha/CR ≥ .70; AVE ≥ .50 |
| 跨文化教学自我效能 ITSE | 6 | Contextualized adaptation based on Romijn et al. (2021) and Deardorff (2006); internationalized/cross-border teaching | 单维 | alpha/CR ≥ .70; AVE ≥ .50 |
| 教学动机：自主动机 ATM | 6 | Adapted from WTMST (Fernet et al., 2008), informed by SDT (Deci & Ryan, 2000); intrinsic and identified regulation | 内在/认同 | alpha/CR ≥ .70; AVE ≥ .50 |
| 职业幸福感 OWB | 6 | Adapted from PERMA-Teacher (Kern et al., 2014) and Warr (1990); meaning, achievement, positive affect, satisfaction | 单维 | alpha/CR ≥ .70; AVE ≥ .50 |
| 情绪耗竭 EE | 5 | Contextualized emotional-exhaustion short form based on MBI-ES (Maslach & Jackson, 1981; Maslach et al., 1996); items were contextualized for this study; the original item wording was not reproduced | 单维 | alpha/CR ≥ .70; AVE ≥ .50 |
| 院校支持感知 PIS | 8 | Contextualized adaptation of POS/SPOS; Eisenberger et al. (1986); AI-supported intercultural/international teaching support | 单维或二阶可检验 | alpha/CR ≥ .70; AVE ≥ .50 |
| 标记变量 MV | 4 | Self-developed neutral work-habit marker for the common-method-bias check | 单维 | alpha ≥ .70 |
| 注意力检测题 | 2 | 自编 | - | - |
| 人口学/控制变量 | 10 | 自编 | - | - |

核心研究测量题合计 47 题；含标记变量 51 题；含人口学和注意力检测共约 63 题。

注：表中阈值为研究设计阶段预设的参考标准。正式结果显示，各构念 AVE 均低于 .50，部分 alpha 低于 .70，详见论文 4.3 节与 5.4 节。 Note: The thresholds are reference criteria specified at the design stage. The observed AVE values were below .50 for all constructs and several alpha values were below .70 (see manuscript Sections 4.3 and 5.4).

# **附录 B：问卷结构说明 Appendix B. Questionnaire Documentation**

**1. 内容效度 Content Validity**

本附录仅记录问卷结构、作答锚点和分析变量对应关系，不新增研究结果或分析结论。

**2. 结构效度与两独立样本 Construct Validity and Two Independent Samples**

样本 A（n = 300）用于 AI 接受度量表的 EFA；样本 B（n = 495）用于 CFA、整体测量模型、结构关联模型和探索性调节分析。两批样本在不重叠的时间段内分别收集；由于问卷匿名，无法在个体层面直接核实两样本无重叠。

**3. 信度与效度 Reliability and Validity**

本研究报告 Cronbach's alpha、组合信度 CR、标准化因子载荷和 AVE；区分效度结果包括 HTMT 矩阵和 AITA 单因子与相关四因子模型比较。

**4. 共同方法偏差 Common Method Bias**

问卷采用匿名作答、题项分区和注意力检测等程序；具体数据处理与统计方法以论文 Methods 为准。

**5. 作答质量 Response Quality**

本研究按照预先设定的质量控制规则筛除无效问卷，包括未通过目标样本筛查、注意力检测题答错、作答时长低于 180 秒，以及核心量表出现明显直线作答。

**6. 语言等值 Language Equivalence**

本问卷以中文正式施测；英文用于论文附录和国际读者理解。题项来源与情境化改编记录见 Appendix A 和论文 Methods。

**7. 量表来源说明 Scale-source documentation**

各构念均在论文 Methods 中注明来源或情境化改编方式；本问卷不重复呈现受版权保护的原始题项。

**8. 计分说明 Scoring**

卷入度（C）和情绪耗竭（H）采用频率计分；其余核心构念采用同意度计分。情绪耗竭得分越高表示损耗越重，预期与职业幸福感呈负向关联。当前版本不设置反向题。

问卷结束，衷心感谢您的参与！

End of questionnaire. Thank you for your participation.

Documentation note: The questionnaire wording and response anchors are aligned with the final analysis codebook; raw numeric responses were unchanged. In the raw survey export, the B8 option “无（None）” was stored as a blank cell; it is coded as None (0) in the analysis codebook and reported as None in Table 1.

Source-record note: The citation is standardized as Romijn et al. (2021).
